# *CRTAP*-Null Osteoblasts Have Increased Proliferation, Protein Secretion, and Skeletal Morphogenesis Gene Expression with Downregulation of Cellular Adhesion

**DOI:** 10.3390/cells14070518

**Published:** 2025-03-31

**Authors:** Aileen M. Barnes, Apratim Mitra, Marianne M. Knue, Alberta Derkyi, An Dang Do, Ryan K. Dale, Joan C. Marini

**Affiliations:** 1 Section on Heritable Disorders of Bone and Extracellular Matrix, *Eunice Kennedy Shriver* National Institute of Child Health and Human Development, NIH, Bethesda, MD 20892, USA; 2 Bioinformatics & Scientific Programming Core, *Eunice Kennedy Shriver* National Institute of Child Health and Human Development, NIH, Bethesda, MD 20892, USA; 3 Office of the Clinical Director, Division of Intramural Research, *Eunice Kennedy Shriver* National Institute of Child Health and Human Development, Bethesda, MD 20892, USA; mmtknue@gmail.com (M.M.K.);

**Keywords:** cartilage-associated protein, osteogenesis imperfecta, 3-hydroylation complex

## Abstract

Type VII osteogenesis imperfecta (OI), caused by recessive *CRTAP* mutations, is predominantly lethal in the first year of life. Due to its early lethality, little is known about bone dysplasia mechanism. RNA-seq analysis of differentiated osteoblasts of siblings with a non-lethal homozygous *CRTAP*-null variant showed an enrichment of gene ontology terms involved in DNA replication and cell cycle compared to control. BrdU incorporation confirmed a ≈2-fold increase in proliferation in non-lethal proband osteoblasts in comparison to control cells. In addition, the expression of cyclin dependent kinase inhibitor 2A (*CDKN2A*), encoding a protein involved in cell cycle inhibition, was significantly reduced (>50%) in *CRTAP*-null osteoblasts, while cyclin B1 (*CCNB1*), encoding a promoter of the cell cycle, was enhanced. Ossification and bone and cartilage development gene ontology pathways were enriched among upregulated genes throughout osteoblast differentiation, as was protein secretion. Ingenuity pathway analysis indicated an upregulation of BMP2 signaling, supported by increase in both *BMP2* and *MSX2*, an early BMP2-responsive gene, by qPCR. Throughout differentiation, *CRTAP*-null osteoblasts showed a decrease in transcripts related to cell adhesion and extracellular matrix organization pathways. We propose that increased proliferation and osteogenesis of type VII OI osteoblasts may be stimulated through upregulation of BMP2 signaling, altering bone homeostasis, and leading to weaker bones.

## 1. Introduction

Osteogenesis imperfecta (OI) is a genetically and clinically heterogeneous collagen-related disorder that is characterized by fragile, bowed bones, and short stature. Autosomal dominant mutations in *COL1A1* or *COL1A2*, the genes encoding type I collagen, account for 80–85% of disease-causing variants [1]. These variants alter the quantity, structure, or processing of the type I collagen protein. Type I collagen is an abundant, crucial, structural component of the extracellular matrix of many tissues, including bones, skin, and tendons. In bones, osteoblasts secrete an abundance of type I collagen into the extracellular matrix, providing the tissue with plasticity, and supplying the framework for the deposited mineral which imbues it with stiffness and strength.

In 2002, Ward et al. described an autosomal recessive form of OI in a First Nations community with moderately severe OI and rhizomelia, localized to chromosome 3p22-24.1 [2], which they termed type VII OI. *CRTAP*, encoding cartilage-associated protein, was discovered to be the underlying genetic cause of type VII OI and was the first recessive OI gene identified [3,4]. CRTAP is a member of the procollagen prolyl 3-hydroxylation complex, which includes prolyl 3-hydroxylase 1 (P3H1) and the *cis-trans* peptidyl-prolyl isomerase B (PPIB), also known as cyclophilin B (CyPB). This complex fully 3-hydroxylates α1 (I) Pro986 and partially hydroxylates α2(I)Pro707 in type I collagen, as well as residues in both types II and V collagen [5]. Subsequently, mutations in both *P3H1* [6] (type VIII OI) and *PPIB* [7] (type IX OI) were also found to cause recessive OI, highlighting the importance of this complex for properly folded collagen. Besides its 3-hydroxylation and prolyl isomerase activity, the 3-hydroxylation complex also functions as a collagen chaperone [8]. The overmodification of collagen proteins only occurs with type I collagen mutations and variants of the 3-hydroxylation complex. CRTAP and P3H1 are especially important members of this complex, as they mutually stabilize each other in the cell [9]. When either protein is absent, the other is degraded leading to the loss of both proteins.

The *Crtap*^−/−^ mouse has provided some insights into potential mechanisms of type VII OI as well as a being a model for severe OI. *Crtap*^−/−^ mice have a severe osteochondrodysplasia, with rhizomelic, osteoporotic bones [4]. A lack of CRTAP leads to a decrease in the bone volume/tissue volume (BV/TV) and a diminished osteoid production. Besides weakening bones, a CRTAP deficiency also affects diverse tissues such as the lungs, kidney, and skin [10], and *Crtap*^−/−^ tendons are thinner and weaker with an increase in irreversible crosslinks [11]. However, individuals with *CRTAP* mutations have a much more severe phenotype than the mouse model, ranging from severe to lethal. Most *CRTAP* mutations cause lethality in the first year of life, with the majority of the surviving *CRTAP* probands having missense, splice-site, or in-frame deletion variants that lead to a diminished amount, rather than complete absence of the CRTAP protein [4,12,13,14,15,16,17,18,19,20,21,22], suggestive of its importance in collagen-containing tissues. *CRTAP* mutations are rare, and in combination with its very severe phenotype, limited bone studies have been conducted. Previous reports have shown that bone samples from *CRTAP* probands have altered osteoid on histology [22] or decreased 3-hydroxylation [14] but osteoblasts have not been available to follow the transcriptional changes occurring throughout differentiation. Here, we present osteoblast and fibroblast data from two siblings with severe type VII OI and *CRTAP*-null mutations, elucidating the osteoblast transcriptional phenotype in this form of OI.

## 2. Materials and Methods

### 2.1. Patient Population

The Institutional Review Board (IRB) of the National Institutes of Health (NIH) approved the protocols (NCT00076830 and NCT03575221). Individuals of various ages and races/ethnicities with bone-related suspected connective tissue disorders or osteogenesis imperfecta were eligible. Evaluations and biospecimen collection were tailored to the participants’ presentation.

### 2.2. Human Subjects and Cell Culture

Osteotomy bone chips and dermal tissues were obtained from surgical discard for patients NL-1 (at age 3 years) and NL-2 (at age 9 years) (*CRTAP* c.[561T > G]; [561T > G], p.[(Tyr187*)]; [(Tyr187*)]) during intermedullary rodding procedures. Control osteoblasts were derived from the surgical discard of a healthy 10-year-old male with skeletal deformities not related to OI (a generous gift of Dr. Michael To). Dermal punch biopsies from newborn patients L-1 (described as infant 2 in Barnes et al. [3]) (*CRTAP* c.[826C > T]; [826C > T], p.[(Gln276*)]; [(Gln276*)]), and L-2 (described as proband 4 in Chang et al. [9])(*CRTAP* c.[826C > T]; [634C > T], p.[(Gln276*)]; [(Arg212*)]) and a healthy individual (aged 5 years) were performed with informed consent under an NIH IRB-approved protocol.

Fibroblast cultures were grown in Dulbecco’s Modified Eagle Medium (DMEM) + GlutaMAX™ (Gibco, Gaithersburg, MD, USA) containing 10% Benchmark™ fetal bovine serum (FBS, Gemini Bio-Products, West Sacramento, CA, USA), and 1% penicillin–streptomycin (Gibco) at 5% CO_2_. Osteoblast primary cultures were established from minced bone chips, using a modified Robey and Termine method [23]. Briefly, bone chips were diced into uniform pieces and digested with Collagenase P (Roche, Basel, Switzerland) at 0.3 U/mL in a serum-free medium for 2 h at 37 °C. Bone chips were plated in Minimum Essential Media alpha (αMEM) containing 10% GemCell™ fetal bovine serum (Gemini Bio-Products), 1% penicillin–streptomycin, and 50 µg/mL ascorbic acid (Sigma-Aldrich, St. Louis, MO, USA) at 8% CO_2_ and cells allowed to outgrow. For differentiation assays, osteoblasts were plated in 6-well plates and switched to differentiation medium (αMEM plus 10% GemCell™ FBS, 1X penicillin-streptomycin, 50 µg/mL ascorbic acid, 2.5 mM beta-glycerophosphate (Sigma), and 10 µM dexamethasone (Sigma)) upon cell confluence and medium was changed thrice weekly. Day 0 osteoblast differentiation samples were collected upon cell confluence but before starting in differentiation medium.

### 2.3. Genomic DNA Preparation and Sequencing

Genomic DNA was prepared from confluent fibroblasts using the Gentra Puregene kit (Qiagen, Hilden, Germany), following the manufacturer’s protocol. Polymerase chain reaction was used to amplify gDNA *CRTAP* intron 1 to intron 2 and sent to Psomagen (Rockville, MD, USA) for the Sanger sequencing of probands and siblings.

### 2.4. Steady State Collagen Biochemical Analysis

Fibroblasts were plated in a 6-well plate and grown to confluency. Cells were serum-starved for 2 h in serum-free DMEM + 50 µg/mL ascorbic acid, then labeled with 260 µCi/mL of 3.96 TBq/mmole L-[2,3,4,5-^3^H]-proline (Moravek, Brea, CA, USA) in the same medium for 18 h. Procollagens were harvested from media and cell layers and precipitated with 1/3 volume of saturated ammonium sulfate. Collagens were pepsin-digested (50 µg/mL) for four hours [24], dried, resuspended in sample buffer (1 M urea, 2% SDS, 18% *w*/*v* glycerol, 0.01% bromophenol blue) and electrophoresed on unreduced 5% SDS-polyacrylamide–urea gels (0.5 M urea) overnight.

### 2.5. RNA Preparation and Real-Time qPCR

Total RNA was harvested from proband and control fibroblasts and osteoblasts using the Qiagen RNeasy^®^ kit (Qiagen), following the manufacturer’s protocol. Samples were first homogenized with QIAshredder columns and included an on-column DNase digestion with an RNase-Free DNase set (Qiagen). Complementary DNA was made using the High Capacity cDNA Reverse Transcription kit (AppliedBiosystems) using RNase Inhibitor (Invitrogen) and used in real-time quantitative PCR with the following Taqman™ probes (*CRTAP*, *P3H1*, *PPIB*, *COL1A1*, *PLOD1*, *ALPL*, *IBSP*, *MEPE*, *SP7*, *CCNB1*, *CDKN2A*, *BMP2*, *MSX2*, *MATN3*, *GREM1*, *GREM2*, *TEK*, *ITGB8*, *CELSR2*, *PCDHGA7*, *PCDHGA12*, *MMP24*, *ICAM1*, *ADAMTSL4*, *ACAN*, and *TJP1*), housekeeping genes (*GAPDH*, *B2M*, and *ACTB*) and Taqman™ Universal Fast PCR Master Mix 2X (#4352042) on a QuantStudio™ 6 Real-time PCR System (AppliedBiosystems) (see Supplementary Table S1 for probe details). qPCR cycling conditions were 95 °C for 20 s, followed by 40 cycles of 95 °C for 1 s, then 60 °C for 20 s. The ΔΔCT method was utilized to calculate the relative expression of the gene of interest compared to the three housekeeping genes (fibroblasts) or to *B2M* (osteoblasts).

### 2.6. RIPA Lysates and Western Blotting

Fibroblast and osteoblast cell lysates were collected in RIPA buffer (150 mM NaCl, 1% NP-40 substitute, 0.5% sodium deoxycholate, 0.1% SDS, 50 mM Tris, pH 8) supplemented with a protease inhibitor cocktail (Sigma–Aldrich, St. Louis, MO, USA) or a protease and phosphatase inhibitor tablet (ThermoFisher, Waltham, MA, USA) from two (osteoblasts) or three (fibroblasts) independently grown cultures. Then, 25 µg of cell lysates were run on 4–15% pre-cast Criterion™ TGX™ midi gels (Bio-Rad, Hercules, CA, USA) and transferred to nitrocellulose using an iBLOT2 (Invitrogen). Blots were blocked in 5% bovine serum albumin (BSA, Gemini BioProducts) in TBST (1X tris-buffered saline + 0.05% Tween-20). Primary and secondary antibodies were diluted in 2.5% BSA/TBST. Blots were visualized on a LI-COR CLx infrared imager (LI-COR, Lincoln, NE, USA). The primary antibodies used were as follows: CRTAP (H00010491-M01, Abnova, Taipei, Taiwan), LEPRE1 (P3H1) MaxPab (H00064175-B01P, Abnova), cyclophilin B (ab16045, Abcam, Cambridge, MA, USA), LH1 (HPA049137, Sigma-Aldrich), alpha-tubulin (05-829, Millipore), BiP (ab32618, Abcam, Waltham, MA, USA), FKBP65 (H00060681-M13, Abnova), HSP47 (ADI-SPA-470-F, Stressgen, Enzo Life Sciences, Farmingdale, NY, USA), Cyclin B1 (122315, Cell Signaling, Danvers, MA, USA), and beta-actin (A5441, Sigma–Aldrich). Secondary antibodies were used at a 1:5000–1:10,000 dilution (IRDye^®^680RD (926-68071) and IRDye^®^800CW (925-32210), LI-COR). LI-COR ImageStudio™ was used to determine the integrated density of proteins inside the regions of interest. The relative levels of each protein were normalized to a loading control (α-tubulin or β-actin) on the same gel. Repeated samples were collected independently then run together for comparison on the same blot.

### 2.7. Electron Microscopy

Osteoblasts were seeded in a 6-well plate and fixed just prior to confluency in glutaraldehyde (2% *v*/*v*) (Tousimis, Rockville, MD, USA)/0.1 M cacodylate buffer (0.1 M, pH 7.4) for 1 h at room temperature, then at 4 °C overnight before storing in PBS in preparation for thin-sectioned EM analysis [25]. The osteoblasts were processed and embedded at room temperature in a fume hood. Following fixation, the osteoblasts were washed 2 times for 10 min in cacodylate buffer before post-fixation of 1 h in osmium tetroxide (1% *v*/*v*, Electron Microscopy Sciences, Ft. Washington, PA, USA). The osteoblasts were then washed 2 times with ddH2O and 1 time with acetate buffer (0.1 M, pH 4.5) before en bloc stain in 0.5% *w*/*v* uranyl acetate (0.5% *v*/*v*) in acetate buffer (0.1 M, pH 4.5) for 1 h. The osteoblasts were dehydrated with multiple washes of EM graded ethanol at 35%, 50%, 75%, 95%, and 100% sequentially. The osteoblasts were then infiltrated with pure epoxy resin (Poly/Bed^®^ 812, Polysciences, Inc., Warrington, PA, USA) overnight. The following day the osteoblasts were washed with pure resin prior to embedding. Immediately after embedding, the 6-well plate was placed in a 55 °C oven to cure for 48 h. Once cured, the resin blocks were separated from the plate and examined under an inverted microscope to select an area with a considerable number of osteoblasts. The preferred area was removed from the block, trimmed, and thin-sectioned using an ultramicrotome (Leica, Bannockburn, IL, USA) equipped with a diamond knife. The thin sections were mounted onto copper mesh grids for counter staining with uranyl acetate and lead citrate. The grids were then carbon-coated in a vacuum evaporator. Once carbon-coated, the grids are prepared to be scanned and imaged. The Hitachi Electron microscope (H7650, Tokyo, Japan) operated at 80 kv with a CCD camera captured the digital images. Electron microscopy was performed at the NCI Electron Microscopy Laboratory core facility (Frederick, MD, USA).

### 2.8. Mass Spectrometry and Amino Acid Analysis

Procollagen prolyl 3-hydroxylation was assessed by ion-trap mass spectrometry, as previously described [3]. Secreted procollagen was precipitated by 1 M (NH_4_)_2_SO_4_, from which proα-chains were resolved by SDS-PAGE and subjected to in-gel trypsin digestion for analysis of the targeted peptides by electrospray mass spectrometry. Amino acid analysis of hydroxylysine and hydroxyproline percentage was performed on conditioned media procollagens by high-pressure liquid chromatography on a Hitachi L8900 Amino Acid Analyzer (AAA Service Lab, Damascus, OR, USA).

### 2.9. Immunofluorescence Microscopy

Fibroblasts or osteoblasts were seeded on 4-well or 8-well chamber slides (Nunc Technologies, Rochester, NY, USA) and incubated under normal growth conditions, until they reached 50–70% confluence. Cells were then grown overnight in growth medium supplemented with 50 µg/mL ascorbic acid for 16 h. After washing briefly with phosphate-buffered saline (PBS), cells were fixed in 4% paraformaldehyde in PBS for 15 min. Cells were permeabilized in 0.1% Triton-X-100 for 15 min on ice, then blocked with 1% BSA in PBS. Primary and secondary antibodies were diluted in 1% BSA in PBS. Cells were incubated with primary antibody for 1 h, washed in 1X PBS, and incubated with an Alexa-fluor secondary antibody solution for 1 h at room temperature. After extensive PBS washes, cells were incubated with 1:5000 dilution of 1 mg/mL DAPI for 10 min, washed again in 1X PBS and coverslipped with Vectashield Plus mounting medium (Vector Laboratories, Burlingame, CA, USA). Cells were examined using a Zeiss LSM 710 scanning confocal microscope (Zeiss Inc., Thornwood, NY, USA) using ZEN operating software at the NICHD Microscopy and Imaging Core (MIC, Bethesda, MD, USA). Antibody dilutions are as follows: Golgin 97 (A-21270, Invitrogen) at 1:200; COL1A1 (LF-68, α1[I] C-telopeptide; a generous gift from Dr. Larry Fisher, NIH) [26] at 1:500; and secondary antibodies Alexa-fluor 488 (A11008) and Alexa-flour 555 (A31570, Thermo-Fisher, Waltham, MA, USA) were used at 1:200.

### 2.10. Collagen Secretion Assay

Cells were plated in triplicate 25 cm^2^ (osteoblasts) or 75 cm^2^ (fibroblasts) flasks, per experiment. When cells were near confluency, they were washed twice in PBS and incubated in serum-free medium for 24 h. Conditioned media were collected with a protease inhibitor cocktail (Sigma) and concentrated with Amicon^®^ Ultra-4 spin columns (Millipore) to a volume of ≤200 µL. Secreted collagen was measured by Sircol™ Assay (biocolor Ltd., Carrickfergus, UK) and normalized by cell number. Cell number was measured for each flask on a DeNovix^®^ CellDrop automated cell counter (Wilmington, DE, USA).

### 2.11. RNA-Seq and Analysis

Triplicate samples from confluent control and patient osteoblasts (day 0) and cells cultured for 7, 14, or 21 days in osteogenic media were analyzed for expression differences between control and both *CRTAP*-null probands by bulk RNA-Seq transcriptomics. RNA quality control was performed on an Agilent 2100 Bioanalyzer (Agilent Technologies, Inc., Santa Clara, CA, USA). One *CRTAP*-null sample, (NL-2, Day 14), was excluded from analysis for degradation (RNA integrity number of 2.5). Poly-A enrichment of total RNA was performed to enhance mRNA transcripts. Then, 2 × 100 bp paired-end reads were performed on an Illumina NovaSeq6000 sequencer (Illumina, San Diego, CA, USA). Reads were assessed for sequencing quality using FastQC v0.11.9 [27], trimmed using cutadapt v3.4 [28], and aligned to the human genome GRCh38 primary assembly using STAR v2.7.8a [29]. Read counts per gene were estimated with featureCounts v2.0.1 [30] using human GENCODE v28 annotation. Mutant samples were compared to wild-type controls separately at each timepoint using DESeq2 v1.28.0 [31] after estimating variances using all samples. Statistical significance was defined based on false discovery rate (FDR) < 0.1 and an absolute log_2_fold-change of at least 1 (2-fold difference). Significantly different genes were analyzed for functional enrichment with clusterProfiler v3.16.0 [32] using the three gene ontologies (Biological Process, Cellular Component, and Molecular Function) and the KEGG pathway database. The RNA-sequencing data have been deposited to the database of Genotypes and Phenotypes (dbGaP) under the accession code phs003969.v1.p1. To access these data, users may apply for access to the dbGaP data repository (http://www.ncbi.nlm.nih.gov/projects/gap/cgi-bin/study.cgi?study_id=phs003969.v1.p1 (accessed on 30 January 2025)). 

### 2.12. BrdU Proliferation Assay

Fibroblasts or osteoblasts were plated in triplicate at 2 × 10^4^ in 96 well plates and treated with BrdU (Abcam, Cambridge, MA, USA) for 24 h. Following the manufacturer’s protocol, cells were fixed, stained, and the absorbance was measured at 450 nm on a VICTOR Nivo™ plate reader (Perkin-Elmer, Waltham, MA, USA).

### 2.13. Statistical Analysis

Statistical significance was determined using unpaired *t*-tests on either Microsoft Excel or GraphPad Prism (v10.0.3). To assess the significance of gene expression during osteoblast differentiation, unpaired *t*-tests were performed for each proband separately vs. control at each timepoint.

## 3. Results

### 3.1. Clinical Report of Female Siblings with Non-Lethal Type VII OI

**Figure 1:**
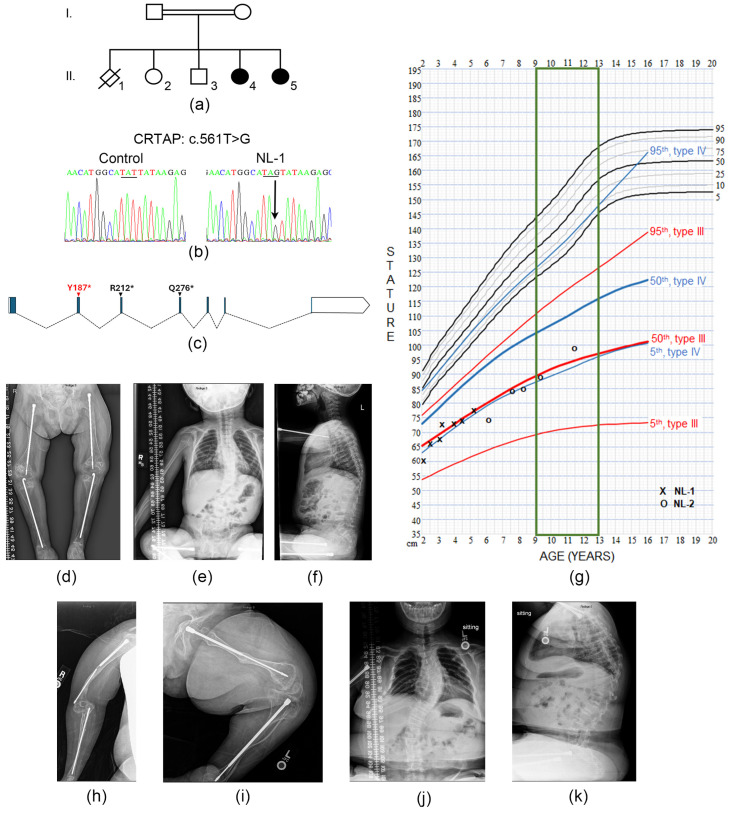
**Radiographs of non-lethal *CRTAP*-null probands** (**a**) Pedigree of the Saudi Arabian family, showing affected individuals in black. Living unaffected siblings were both confirmed to have a normal *CRTAP* sequence; parents are obligatory heterozygotes. In the pedigree, Roman numerals represent familial generations, while numbers across each generation represent the order of birth. Filled shapes denote the affected siblings. (**b**) Sequence tracings showing the homozygous c.561T > G; p.(Tyr187*) mutation in proband NL-1 fibroblasts, leading to a stop codon. Colors represent the different nucleotides of the sequence; the underline highlights the amino acid codon change from a TAT>TAG. (**c**) Schematic of *CRTAP* RNA structure showing the location of the NL (red) and L (black) proband mutations. (**d**–**f**) Radiographs of proband NL-1 at 6 years and 5 months of age. (**d**) Lower long bone radiograph shows bilateral rodding of femora and tibiae with bulbous epiphyses. (**e**) AP spine radiographs showing mild scoliosis. (**f**) Lateral spine radiographs reveal vertebral compressions. (**g**) OI-specific growth curves reveal both NL-1 and NL-2 have a growth curve in the 50th percentile for type III OI children. (**h**–**k**) Radiographs of proband NL-2 at 12 years and 7 months of age. (**h**) Upper extremity radiograph showing broken humeral rod and bulbous epiphyses. (**i**) Lower long bone radiograph reveals rodding of femur and tibia with thin cortices and bulbous epiphyses. (**j**) AP spine radiograph shows moderate scoliosis. (**k**) Lateral spine radiograph shows concave vertebral bodies.

Proband 1 (NL-1) is an 8-year-old girl at the time of this report, born to consanguineous first cousin parents from Saudi Arabia (Figure 1a, II.5). She is the fifth child of five live births with two older living healthy siblings, one sibling deceased at 2 months and an older sister who also has OI. She was born to a G5P4 mother at term via cesarean section for malpresentation. She weighed 2.77 kg (<3rd centile) at birth and had 18 fractures. She was intubated for 5 days and received intensive care for 3 weeks after birth. She was confirmed to be homozygous for the same *CRTAP* variant (NM_006371.5: c.[561T > G]; [561T > G], NP_006362.1: p.[(Tyr187*)]; [(Tyr187*)]) that was first identified in her older sibling (Figure 1b).

During the first 2 years of life, she experienced multiple fractures, including at least three femur fractures. She had bowing of the femora and tibia bilaterally and L humerus and radius, as well as 12th rib agenesis. After her second birthday, she began a series of intramedullary roddings of both the upper and lower extremities (Figure 1c). She developed moderate scoliosis, with compressions of all thoracic and lumbar vertebrae (Figure 1d,e) and “popcorn” deformities of distal femoral and proximal tibial metaphyses, bilaterally. At age 3 years, her L1-L4 DXA z-score = −7.3. Her growth has always been severely impaired, tracking just over the 50% length curve for type III OI (Figure 1f). At age 8 years, her height is 83 cm (50th centile for 21-month-old girl without OI).

Evaluations at the NIH Clinical Center detected mild conductive hearing loss bilaterally at age 6 years and 5 months. Intermittent esotropia and thin cornea were noted at 5 years and 0 months. Cranial CT at 3 years and 11 months detected brachycephaly, Wormian bones, and platybasia without basilar invagination. Her echocardiogram was normal. Pulmonary function tests showed no restrictive or obstructive patterns on the spirometry. Chest CT showed stable scarring in R upper lobe; there was no history of significant respiratory illnesses, such as pneumonia. Bone-related serum assays were normal except for elevated osteocalcin (peak value 58.4 ng/mL, reference range 7.3–38.5 ng/mL), reflecting the high turnover bone metabolism of type VII OI. Developmental assessments showed delays in all areas, especially motor functions. She first pulled to stand at age 5 years. She has received 1 mg/kg pamidronate iv every 3 months since day 3 of life.

Proband 2 (NL-2) is a 14 years and 2-months-old girl with type VII OI at the time of this report. She is the older sister of Proband 1 (Figure 1a, II.4). She was born in Saudi Arabia at term to a G4P3 mother and delivered via cesarean section secondary to finding 15–18 fractures on a pre-natal ultrasound. At birth, she weighed 2.29 kg (<3rd centile) and had fractures in the left clavicle and femur and right tibia/fibula. She was diagnosed with Osteogenesis Imperfecta type VII in Saudi Arabia [33] prior to emigrating to the US with her parents soon after her birth for further care. Genetic testing identified a homozygous *CRTAP* variant, c.561T > G; p.(Tyr187*).

During the first year of life, she sustained 15–20 long bone fractures, including at least four femur fractures. There was bowing of the radius bilaterally, hip laxity, and pes planus. Skeletal radiographs showed undertubulated long bones, and compression of vertebrae throughout the thoracolumbar spine. She has a barrel chest and mild kyphoscoliosis. Radiographs showed “popcorn” metaphyses of the lower extremities. She underwent intramedullary rodding of the femora and tibiae bilaterally around age 2 years, and of bilateral humerii and radii at age 3 years, with multiple subsequent rod revisions and more than 25 fractures. At six years of age, her L1-L4 DXA z-score = −4.17. She has had consistently severe growth deficiency, with a length curve between the 50 and 75th centile of length curve for type III OI (Figure 1f). Her current length of 98 cm is the 50th centile for a 3-year-old girl without OI.

Examinations at the NIH Clinical Center revealed mild bilateral conductive hearing loss. She had refractive amblyopia requiring corrective lenses. Cranial CT showed Wormian bones without platybasia or basilar invagination and an enlarged CSF space without hydrocephalus. A mildly dilated ascending aorta and bicuspid aortic valve were found on an echocardiogram. Chest CT showed local scarring and ground glass nodules [34]. On spirometry assessment, a moderate to severe restrictive defect and mild lower airway obstruction were noted. She has a history of chronic aspiration and reflux. Bone-related serum values were normal except for an elevated osteocalcin of 52.5 ng/mL, reflecting the high turnover bone metabolism of type VII OI. Her motor development was severely impaired. She sat at age 2 years, never pulled to stand, and now uses an electric wheelchair for mobility. Cognitive and social development were age appropriate, and she excels at school.

She was started on 1 mg/kg IV pamidronate on day 20 of life and has continued to receive infusions every 3 months since establishing care in the US. Current radiographs show thin cortices on the femora with bulbous epiphyses, mild scoliosis, and vertebral compressions (Figure 1g–j). An extended clinical report on the siblings is available in the Supplementary Information.

Clinical reports of proband L-1 (described as infant 2 in Barnes et al. [3]), with *CRTAP* c.[826C > T]; [826C > T], p.[(Gln276*)]; [(Gln276*)] and proband L-2 (described as proband 4 in Chang et al. [9]), with *CRTAP* c.[826C > T]; [634C > T] p.[(Gln276*)]; [(Arg212*)] were previously published.

### 3.2. Fibroblasts from Non-Lethal and Lethal CRTAP-Null Patients Have Similar Chaperone Profiles

**Figure 2:**
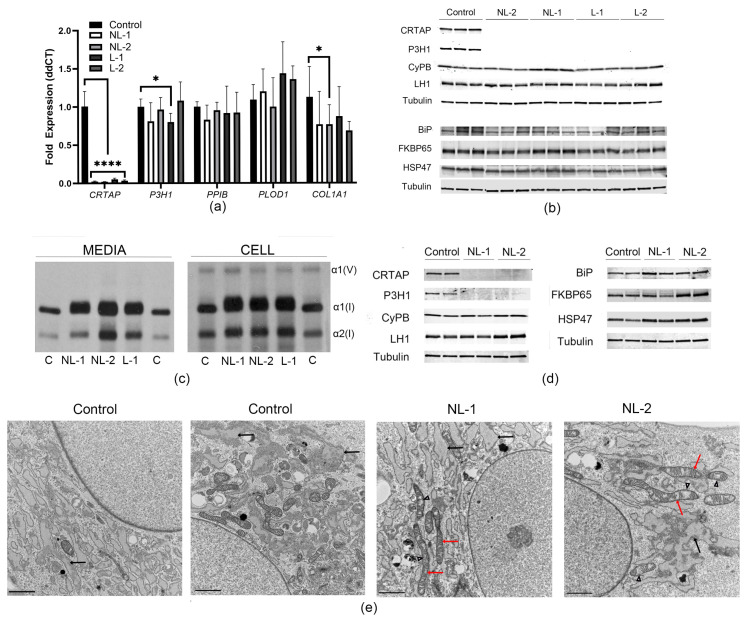
**Non-lethal and lethal *CRTAP*-null probands have similar fibroblast characteristics.** (**a**) *CRTAP* expression in fibroblasts was 2–6% of control fibroblasts in both non-lethal and lethal *CRTAP* proband fibroblasts. There were no other consistent changes in expression in collagen-related genes *P3H1*, *PPIB*, *PLOD1*, and *COL1A1* between lethal and non-lethal probands (n = 4–6). *p*-values were determined using an unpaired *t*-test compared to control, error bars represent ± SEM (* *p* < 0.05, **** *p* < 0.0001). (**b**) Western blotting of collagen-related proteins in *CRTAP* proband fibroblasts show that all lack CRTAP and P3H1 protein expression with no significant changes in PPIB, LH1, BiP, and HSP47. A separate tubulin-loading control is shown for each set of proteins blotted on the same membrane (n = 3–6). (**c**) Migration of α1(I) and α2(I) collagen bands shown by incorporation of ^3^H-proline and separation by unreduced 5% SDS-Urea-PAGE. The migration of collagen from fibroblasts of non-lethal patients is similar to collagen from fibroblasts from lethal *CRTAP* proband L-1. Collagen shows full overmodification of α1(I) and α2(I) collagen chains in all three patients (n = 1–2). (**d**) Western blotting of collagen-related proteins in *CRTAP* proband osteoblasts shows similar trends as in fibroblasts for CRTAP, P3H1, and CyPB. A significant increase in HSP47 protein is the only difference in both *CRTAP* non-lethal probands compared to control, while NL-2 trends towards increased FKBP65 and LH1 protein levels compared to control. BiP levels were elevated in both *CRTAP*-null individuals compared to control but did not reach significance (*p* = 0.2). A separate tubulin-loading control is shown for each set of proteins blotted on the same membrane (n = 4). (**e**) Transmission electron micrographs show that control and proband osteoblasts have mildly dilated endoplasmic reticulum (black arrows). *CRTAP*-null probands both have elongated mitochondria (red arrows) with some areas of mitochondrial cristolysis (arrowheads). Scale bar = 2 µm.

Since *CRTAP*-null mutations usually cause lethal OI, we examined whether there were any differences in collagen migration, chaperone, or transcript levels in fibroblasts from our non-lethal (NL) compared to lethal (L) *CRTAP*-null probands. In agreement with a null mutation, *CRTAP* expression was 0.024 ± 0.01 and 0.021 ± 0.00 in NL-1 and NL-2, respectively, compared to 0.052 ± 0.02 in L-1 and 0.036 ± 0.01 in L-2 when expression in control fibroblasts was set at 1 (Figure 2a). Expression of collagen and collagen-related genes (*COL1A1*, *P3H1*, *PPIB*, and *PLOD1*) were unchanged between NL and L probands and not significantly different from control as a group (Figure 2a). Likewise, protein levels of CRTAP and P3H1 were completely absent in all probands, and collagen chaperones cyclophilin B, lysyl hydroxylase 1 (LH1), BiP, FKBP65, and HSP47 were not significantly different between NL and L fibroblasts (Figure 2b). The migration of collagen α1(I) and α2(I) chains was broadened by excess overmodification of collagen chains and had a similarly delayed migration in both NL and L probands (Figure 2c).

In *CRTAP*-null osteoblasts, NL-2 had increased levels of both FKBP65 (2-fold) and LH1 (1.3-fold) compared to control, but protein levels were normal in NL-1, suggesting it is not part of the NL *CRTAP*-null phenotype (Figure 2d). However, the levels of the collagen chaperone HSP47 were slightly, but significantly increased by 22% (*p* = 0.02) in both non-lethal proband osteoblasts compared to control (Figure 2d). An increase in collagen chaperones may indicate that collagen is not being efficiently trafficked through the cell. On electron microscopy, the endoplasmic reticulum in control, NL-1, and NL-2 osteoblasts appeared slightly dilated in some areas of the cell, likely due to the secretory nature of these cells (Figure 2e). Notably, some of the mitochondria in both NL siblings appeared lengthened and enlarged with mild cristolysis as compared with the control.

The absence of CRTAP leads to the destabilization of P3H1; thus, it is expected that a CRTAP deficiency would lead to diminished type I collagen 3-hydroxylation. Missense and splice variants of *CRTAP* are generally milder in phenotype and have been shown to retain residual levels of Pro986 3-hydroxylation [13,14,22]. Mass spec analysis of secreted collagen from our two NL probands shows that 3-hydroxylation is absent or at very low levels in both fibroblasts and osteoblasts (Table 1), similar to the results we had previously shown for the lethal *CRTAP* probands [3,9].

### 3.3. Collagen Protein Trafficking Is Delayed

**Figure 3:**
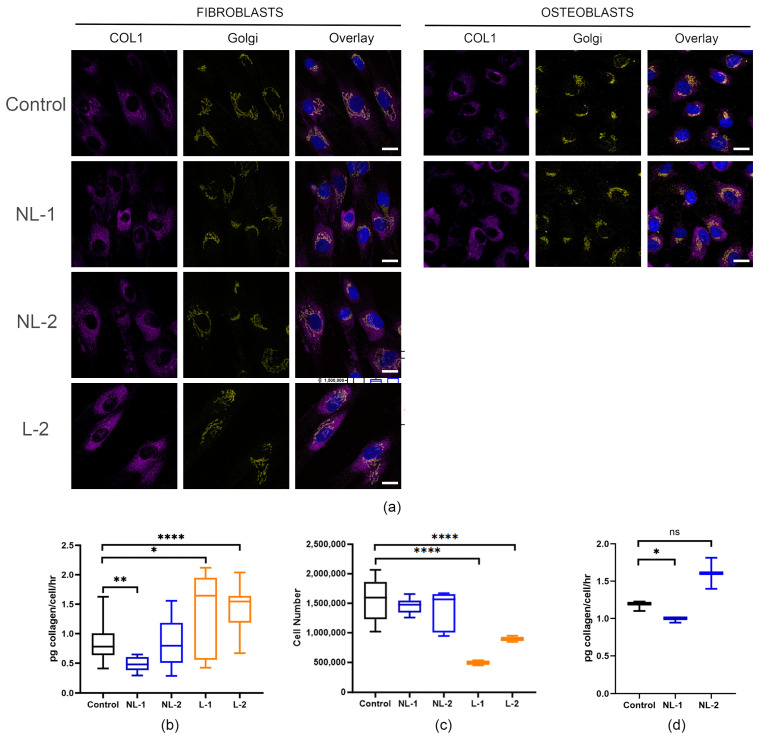
***CRTAP*-null cells have altered collagen trafficking.** (**a**) Immunofluorescence microscopy reveals an increase in collagen-staining (purple) that is not colocalized with the Golgi (yellow) in *CRTAP*-null proband fibroblasts and osteoblasts after overnight stimulation with ascorbic acid. The lethal type VII proband (L-2) appears to have a higher amount of collagen retained in the ER as compared to the non-lethal probands. In osteoblasts, there is a similar trend of nearly full overlap of collagen and Golgi (coral color on overlay panels) in control osteoblasts and retention of collagen in the ER in *CRTAP*-null proband osteoblasts (scale bars = 20 µM, n = 2). (**b**) Sircol assay of collagen secretion shows normal to decreased secretion in non-lethal *CRTAP*-null fibroblasts (blue), with increased collagen secretion per cell in lethal proband fibroblasts (orange) (n = 3–5, 2–3 flasks per experiment). (**c**) Cells counted by an automated cell counter show that fibroblasts from non-lethal patients maintain a normal cell count, while fibroblasts from lethal *CRTAP* probands L-1 and L-2 have a decreased cell count. (**d**) Collagen secretion in *CRTAP*-null osteoblasts NL-1 and NL-2 displays a similar trend to their respective collagen secretion in fibroblasts (n = 1, 3 flasks each). *p*-values were determined using an unpaired *t*-test compared to control, error bars represent ± SEM (* *p* < 0.05, ** *p* < 0.01, **** *p* < 0.001, ns = not significant).

As type I collagen protein in *CRTAP*-null individuals is post-translationally overmodified, we hypothesized that this would lead to delayed collagen trafficking through the endoplasmic reticulum (ER) and Golgi networks. Therefore, we treated cells with ascorbic acid overnight to stimulate collagen production and secretion. In control cells, the collagen signal (yellow) overlaps with the Golgi marker Golgin-97 (purple, with overlap in coral, Figure 3a), confirming normal collagen trafficking. In contrast, collagen localization in both NL and L fibroblasts is partially retained in the endoplasmic reticulum outside the Golgi, suggesting that it is not well trafficked through the secretory pathway. This altered collagen localization is also seen in *CRTAP*-null osteoblasts. Fibroblasts from one lethal *CRTAP*-null individual appeared to have increased collagen retention in the endoplasmic reticulum compared to control and cells from NL individuals.

Fibroblasts from *CRTAP* probands have previously been shown to deposit less collagen in the extracellular matrix [35]. Since the secretion of collagen from the cell would limit how much collagen was ultimately deposited in the matrix, we looked at the collagen secretion rate in both *CRTAP*-null fibroblasts and osteoblasts compared to the controls. Like *P3H1/LEPRE1*-null probands [6], lethal *CRTAP* probands have an approximately 55–65% increase in collagen secretion per cell (Figure 3b). Surprisingly, the NL probands did not follow this trend, but instead have a decreased (NL-1) or normal (NL-2) amount of collagen secreted. We observed that fibroblasts from lethal *CRTAP*-null probands consistently had a lower cell count, with cells attaining confluency at a lower cell number (Figure 3c), which would affect how much collagen was secreted on a per cell basis. This cell count is a steady-state measurement of the number of cells at confluency and does not provide information on whether these changes are reflective of decreased proliferative capacity or increased amount of cell death. The amount of total collagen secreted in 24 h by cells with non-lethal and lethal *CRTAP* variants is similar; however, when normalized to cell count, the secretion rate of the lethal variants appears increased (Figure 3b). The osteoblasts from the NL probands show a similar trend of collagen secretion to their fibroblasts, with a decreased amount of collagen secretion per cell in NL-1 osteoblasts and normal collagen secretion in NL-2 osteoblasts (Figure 3d).

### 3.4. Upregulation of Cell Cycle Genes in CRTAP-Null Osteoblasts

**Figure 4:**
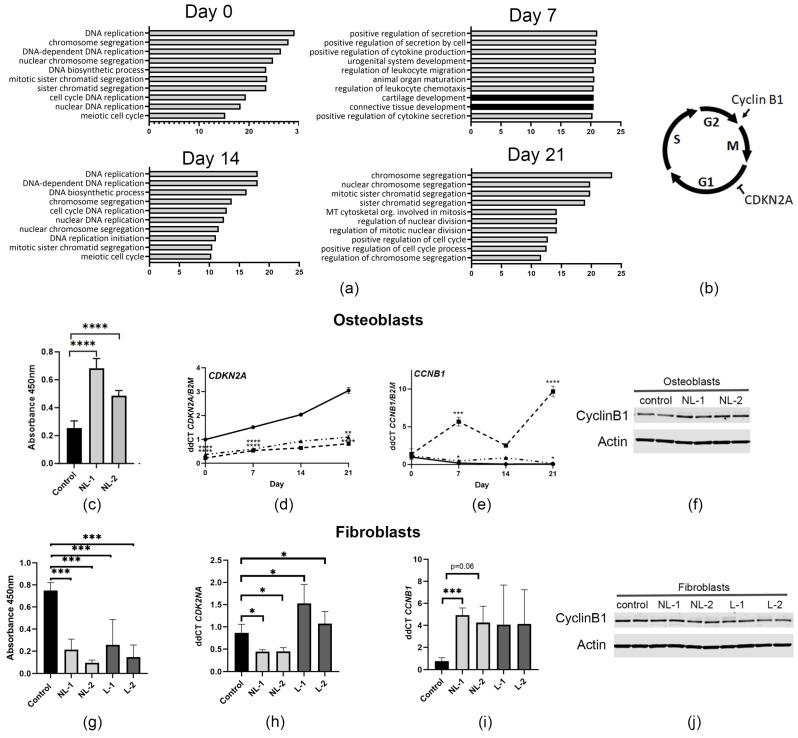
**Proliferation is upregulated in *CRTAP*-null osteoblasts.** (**a**) The top ten gene ontology (GO) biological processes enriched in genes upregulated in *CRTAP*-null proband osteoblasts compared to control throughout osteoblast differentiation were almost all related to the cell cycle, cell division and DNA synthesis. Black bars signify pathways involved in cartilage and connective tissue development. The x-axis depicts −log10 *p*-value. (**b**) Schematic diagram showing the phase in which Cyclin B1 and cyclin-dependent kinase inhibitor 2A (CDKN2A) affect the cell cycle. (**c**) BrdU proliferation assay confirmed increased proliferation in undifferentiated *CRTAP*-null osteoblasts (n = 2, using 3–4 independent samples). (**d**,**e**) Gene expression levels of control (solid black line), NL-1 (dashed line), and NL-2 (dot-and-dashed line) osteoblasts at various timepoints of osteoblast differentiation. (**d**) *CDKN2A* expression is significantly decreased in differentiated *CRTAP*-null osteoblasts (n = 3). (**e**) *CCNB1* expression is increased in differentiated *CRTAP*-null osteoblasts (n = 3). (**f**) Undifferentiated *CRTAP*-null osteoblasts have a slight increase in cyclin B1 protein levels which did not reach significance (~30% increase, *p* = 0.15, n = 2). (**g**) *CRTAP*-null fibroblast proliferation rate is decreased in both non-lethal and lethal *CRTAP* cells (n = 4). (**h**) Non-lethal *CRTAP* proband fibroblasts have decreased expression of *CDKN2A* (~50%, *p* < 0.05), while there is an increase in expression in lethal *CRTAP*-null fibroblasts (24–77%, *p* < 0.05, n = 3). (**i**) *CCNB1* expression is increased in fibroblasts from non-lethal *CRTAP*-null patient NL-1 and trended toward an increase in fibroblasts from NL-2 (*p* = 0.06) and lethal *CRTAP*-null individuals as compared to control (*p* = 0.2–0.3, n = 3). (**j**) There is a slight decrease in cyclin B1 protein levels in *CRTAP*-null fibroblasts (~20% decrease, *p* = 0.0049, n = 2). *p*-values were determined using an unpaired *t*-test compared to control. Error bars represent ± SEM (* *p* < 0.05, ** *p* < 0.01, *** *p* < 0.005, **** *p* < 0.001).

Although *CRTAP* mutations were first identified in 2006 [4,7], gene expression in *CRTAP*-null osteoblasts has yet to be studied in detail. Osteoblast cultures from both NL siblings allowed us to perform an RNA-seq analysis on primary undifferentiated (day 0) and differentiated (day 7, 14, and 21) cells to delineate changes in gene expression during osteoblast maturation in type VII OI (Supplementary Table S2). Surprisingly, we found that the topmost upregulated biological processes in *CRTAP*-null osteoblasts at all timepoints involved DNA replication and the cell cycle (Figure 4a).

The cell cycle is regulated by various stimulators and inhibitors of the different phases of the cell cycle (Figure 4b). First, to determine whether this upregulation of cell cycle and replication genes affected osteoblast proliferation, subconfluent undifferented osteoblasts were incubated with BrdU for 24 h. Osteoblast proliferation of the *CRTAP*-null siblings was significantly increased by 1.9–2.7-fold (*p* < 0.0001), compared to control osteoblasts (Figure 4c). Two cell cycle genes that are involved with increased proliferation and are differentially expressed in the RNA-Seq data were examined in further detail: *CDKN2A*, which encodes cyclin-dependent kinase inhibitor 2A, and *CCNB1*, encoding cyclin B1. *CDKN2A* is a negative regulator of proliferation, inhibiting the cell cycle at the G1 phase, while cyclin B1 promotes G2 to M phase progression [36,37]. In the *CRTAP*-null osteoblasts, levels of *CDKN2A* transcripts were less than half of the control (Figure 4d). Conversely, *CCNB1* transcripts were increased in the null cells (Figure 4e). On western blot, Cyclin B1 protein levels trended higher (23–34%) in *CRTAP*-null osteoblasts, although they did not reach significance (*p* = 0.15) (Figure 4f). These trends in cell cycle proteins correlate with the increase in osteoblast proliferation.

To ascertain whether the increased proliferation was specific to osteoblasts or reflected a global pattern of increased propagation, we examined proliferation in type VII OI fibroblasts. *CRTAP*-null fibroblasts, however, did not follow the same proliferation trends as type VII osteoblasts. BrdU staining revealed that the proliferation was decreased in all *CRTAP*-null fibroblasts, both lethal and non-lethal (Figure 4g). This was unexpected, as the lethal cells were larger in size and had fewer cells at confluency (Figure 3c), while fibroblasts from non-lethal *CRTAP*-null probands were more similar in appearance to control cells. The cells from the non-lethal siblings maintained significantly lower *CDKN2A* levels throughout differentiation, compared to both lethal probands and control fibroblasts as well as increased *CCNB1* levels (Figure 4h,i). In contrast to the transcript levels, cyclin B1 protein levels were significantly decreased by 20% in type VII fibroblasts (*p* = 0.005), with no difference between protein levels from non-lethal and lethal patients (Figure 4j).

### 3.5. CRTAP-Null Osteoblasts Upregulate Protein Secretion Early in Differentiation

After seven days of culture in osteogenic medium, genes involved in protein secretion are upregulated in *CRTAP*-null osteoblasts (Table 2). Cytokine and interleukin secretion pathways were upregulated, suggesting a potential inflammatory response. Insulin-like Growth Factor (IGF-1), which stimulates bone growth by promoting osteoblast differentiation and proliferation, was significantly upregulated starting at differentiation day 7 (log_2_fold = +2.57, *p*-adj = 3.468e^−8^). Many of the genes regulating secretion processes (*FGR*, *INHBB*, *NPY2R*, *C1QTNF3*, *F2RL1*, and *TSLP*), including the regulation of hormone secretion, start to become elevated at day 7 and remain elevated throughout osteoblast differentiation (Supplementary Table S2).

### 3.6. Upregulation of Cartilage and Skeletal Developmental Genes

**Figure 5:**
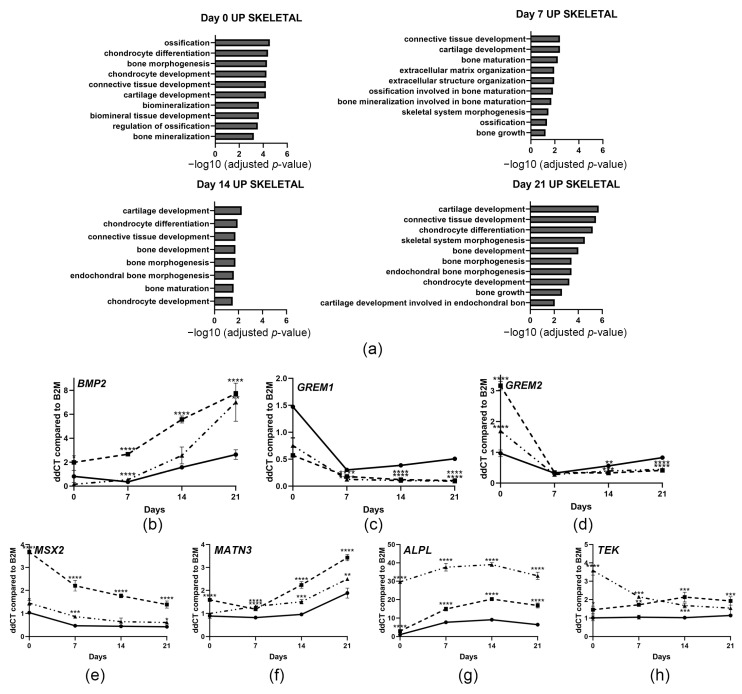
**Upregulation of chondrocyte differentiation and BMP2-related genes.** (**a**) Gene ontology (GO) biological processes involved in cartilage and skeletal development enriched in genes upregulated in *CRTAP*-null osteoblasts during osteoblast differentiation. (**b**–**h**) Gene expression levels of control (solid black line), NL-1 (dashed line), and NL-2 (dot-and-dashed line) osteoblasts at various timepoints of osteoblast differentiation (n = 3). (**b**) *BMP2* transcripts are increased in differentiated osteoblasts from both *CRTAP*-null individuals starting at day 7 of differentiation, compared to control. (**c**,**d**) There is a downregulation of BMP2 antagonists *GREM1* and *GREM2* expression in differentiated osteoblasts concurrent with the increase in *BMP2* transcripts. (**e**,**f**) Cartilage differentiation genes Msh homeobox 2 (*MSX2*) and matrilin-3 (*MATN3*) are significantly increased in differentiated osteoblasts of both *CRTAP*-null siblings compared to control. (**g**) Early osteoblastic marker alkaline phosphatase (*ALPL*) is increased in *CRTAP*-null proband differentiated osteoblasts. (**h**) Endothelial tyrosine kinase receptor (TEK/TIE2) is elevated throughout *CRTAP*-null osteoblast differentiation. *p*-values were determined using an unpaired *t*-test compared to control. Error bars represent ± SEM (* *p* < 0.05, ** *p* < 0.01, *** *p* < 0.005, **** *p* < 0.001).

Osteoblast and cartilage-related pathways such as skeletal system morphogenesis, ossification, chondrocyte differentiation, and connective tissue development were also significantly enriched in genes upregulated in *CRTAP*-null osteoblasts, though the level of statistical significance is not as high as for cell cycle pathways (Figure 5a, Supplementary Tables S2 and S3). RNA-sequencing revealed that many genes in BMP2 signaling pathways were differentially expressed. BMP2 signaling is involved in osteoblast differentiation and cartilage development and thus changes in signaling may greatly affect bone properties. Quantitative PCR confirmed that *BMP2* expression is increased in *CRTAP*-null osteoblasts during differentiation, while BMP2 antagonists *GREM1* and *GREM2* have decreased transcripts (Figure 5b–d). *MSX2* and *MATN3* genes promoting cartilage differentiation [38,39,40] are significantly up in both probands throughout the osteoblast timecourse (Figure 5e,f). In addition, genes involved in skeletal morphogenesis such as alkaline phosphatase (*ALPL*) and the TEK receptor tyrosine kinase (*TEK*/*TIE2*) were substantially increased (Figure 5g,h).

### 3.7. Downregulation of Genes Involved in Cell Adhesion and Extracellular Matrix Organization

**Figure 6:**
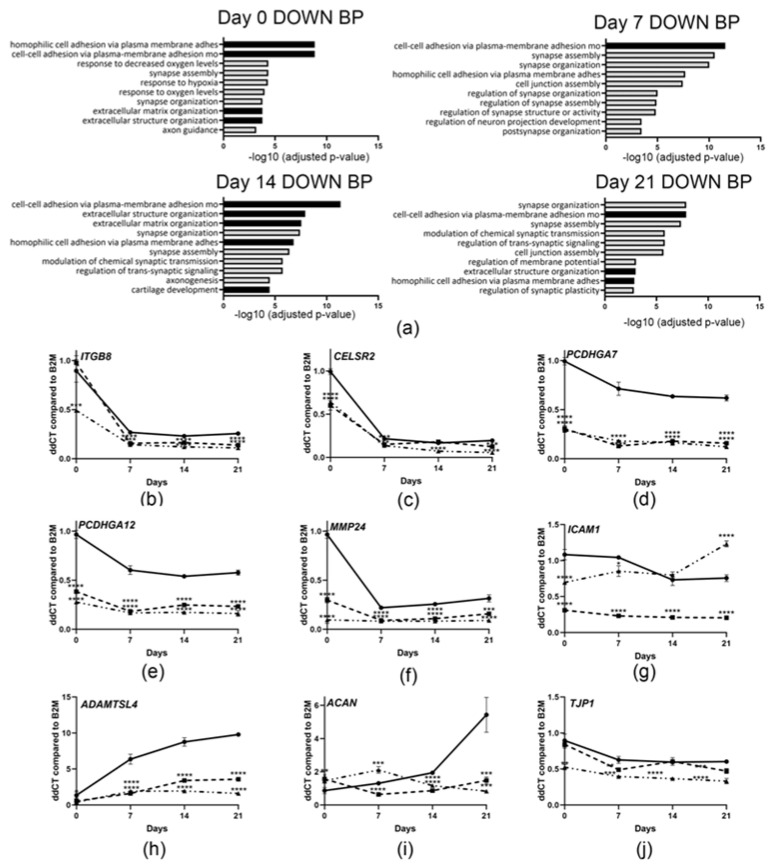
**Cell adhesion genes are downregulated in *CRTAP*-null differentiated osteoblasts.** (**a**) Functional enrichment analysis showing the top ten downregulated biological processes in *CRTAP*-null osteoblasts at each differentiation timepoint. Black bars signify pathways involved in cell adhesion or extracellular matrix. (**b**–**j**) Gene expression levels of control (solid black line), NL-1 (dashed line), and NL-2 (dot-and-dashed line) osteoblasts at various timepoint of osteoblast differentiation (n = 3). (**b**) Expression of integrin beta 8 (*ITGB8*) was decreased in differentiated *CRTAP*-null osteoblasts. Cadherin related transcripts (**c**) *CESLR2*, (**d**) *PCDHGA7*, (**e**) *PCDHGA12*, and (**f**) *MMP24* were decreased at all timepoints of differentiation. *CRTAP*-null differentiated osteoblasts had decreased expression of cellular adhesion transcripts for (**g**) intracellular adhesion molecule 1 (*ICAM1*), (**h**) metalloproteinase ADAMTS like 4 (*ADAMTSL4*), (**i**) aggrecan (*ACAN*), and (**j**) tight junction protein-1 (*TJP1*). *p*-values were determined using an unpaired *t*-test compared to control. Error bars represent ± SEM (* *p* < 0.05, ** *p* < 0.01, *** *p* < 0.005, **** *p* < 0.001).

In classical osteogenesis imperfecta, type I collagen is decreased in amount or has an altered structure. Although *CRTAP*-null mutations have been shown to increase the post-translational modification of collagen [3] and have a deficiency of collagen in the extracellular matrix [35], it is unclear how this might affect gene transcription. In our RNA-Seq analysis, we found that *CRTAP*-null osteoblasts had decreased cell adhesion transcripts throughout the osteoblast differentiation timecourse (Figure 6a), suggesting that it is a consistent feature of type VII OI. Furthermore, GO terms related to the organization of the extracellular matrix, chondrogenesis (Supplementary Table S4), and axonogenesis are also enriched in downregulated genes.

A variety of proteins are involved in cell–cell adhesion, connecting the cells to each other and to the extracellular matrix. *CRTAP*-null osteoblasts have decreased the expression of many types of cell adhesion molecules, suggesting that a global decrease in adhesion transcripts may lead to a decrease in cellular adhesion. Integrins are cell surface receptors which connect cells to extracellular matrix proteins. Osteoblasts from the *CRTAP*-null siblings show a significant decrease in expression of integrin β8 (*ITGB8*, Figure 6b), which is also a mediator of latent TGF-β activation [41]. Transcripts of cadherin proteins, which are involved in cell–cell adhesion, are also downregulated, including the cadherin receptor (*CELSR2*), gamma-protocadherins (*PCDHGA7* and *PCDHGA12*) and *MMP24*, which encodes an enzyme that cleaves cadherins (Figure 6c–f). *ICAM1* and *ADAMTSL4*, which encode glycoproteins that bind to actin and fibrillin-1, respectively, and *ACAN*, encoding the cartilage proteoglycan aggrecan, are also decreased (Figure 6g–i), suggesting that the composition of the extracellular matrix is altered. Lastly, tight junctions, composed of adhesion proteins, are important for exchanging molecules between cells. ZO-1/*TJP1* is also downregulated in *CRTAP*-null osteoblasts (Figure 6j) which may affect tight junction stabilization to the actin cytoskeleton.

## 4. Discussion

Here, we present the first characterization of gene expression pathways in human *CRTAP*-null osteoblasts. Previous studies of individuals with variants in *CRTAP* have mainly focused on mutational detection and function, as well as clinical descriptions. Using osteoblasts obtained from non-lethal *CRTAP*-null siblings, we have revealed signaling pathways enriched in type VII OI. Along with an increase in osteoblastic gene expression, we also found a surprising elevation of DNA replication transcripts and a decrease in cell adhesion expression contributing to the phenotype of these patients. Although the rarity of patients with type VII OI who survive beyond infancy limited this study to the information that we could obtain from two patients’ osteoblasts, we anticipate that this study can provide a framework for better understanding of the disease mechanism.

Many of the *CRTAP* variants reported in the literature are from large patient screenings to identify OI-causing mutations. These reports mainly consist of clinical descriptions of the individuals with their identified mutation [15,17,20,33]. Although *CRTAP* mutations were first identified more than a decade ago, little is known about the cellular aspect of the disease mechanism in type VII OI, besides a decrease in *CRTAP* transcripts, CRTAP and P3H1 protein, and a lack of 3-hydroxylation. We previously showed that CRTAP deficiency decreases the amount of collagen matrix deposited in vitro [35]. Additionally, a study of the bone calcium content from an individual with a hypomorphic *CRTAP* mutation showed that the *CRTAP*-deficient bone has a similar level of bone hypermineralization as other classical OI types caused by collagen defects [42]. Since *CRTAP* mutations were first identified, bone histology examinations of variable extent have been reported from three individuals, displaying a range of outcomes. Bone histology from an individual from the Canadian First Nations community with hypomorphic type VII, revealed increased osteoid volume, bone formation, and turnover [2]. An individual with a homozygous *CRTAP* single nucleotide deletion, leading to a null outcome, showed woven bone and hypercellular trabeculae [21], while bone histology from an individual with compound *CRTAP* splice mutations and no detectable CRTAP protein had decreased osteoid and bone formation [22].

The presence of misfolded or overmodified collagens can cause ER stress or protein aggregation and lead to induction of the unfolded protein response (UPR). The UPR stimulates an increase in BiP (also known as GRP78) and other chaperones to bolster cell survival. We saw no change in BiP, or other collagen chaperone transcripts or protein levels in fibroblasts from our *CRTAP*-null patients and only a slight increase in HSP47 protein levels in their osteoblasts, suggesting that ER stress does not play a major role in the cellular phenotype. In agreement with this, we do not see an increase in dilated ER in either *CRTAP*-null osteoblasts compared to control. Additionally, the extent of overmodification of collagen chains on collagen biochemistry was similar between cells from non-lethal and lethal patients. Both types of mutations show retention of collagen in the ER on immunofluorescence microscopy, though more collagen appears to be retained in fibroblasts from patients with lethal type VII OI as compared to the non-lethal patients. The decreased ability to traffic collagen into the extracellular matrix may be a contributing factor to these individuals’ early lethality but is unlikely to be the decisive factor since a 50% decrease in structurally normal collagen causes mild OI.

One difference between fibroblasts from lethal and non-lethal individuals was an increase in the calculated collagen secretion rate per cell in lethal probands. Similarly, increased collagen secreted per cell was also reported in P3H1-deficient cells when measured by a pulse–chase assay [6]. For the cells from probands with lethal type VII OI, cell size appeared larger in vitro on visual inspection of flasks, supported by a lower number of total cells at confluence. Another difference is that fibroblasts from patients with lethal type VII OI had a normal level of *CDKN2A* transcripts, while fibroblasts from individuals with non-lethal type VII OI had a significantly lower expression level of the cell cycle inhibitor. The decrease in CDKN2A may allow the non-lethal cells to proliferate despite the presence of retained cellular collagen, as misfolded protein has been shown to be able to inhibit proliferation in COS-7 cells [43]. In osteoblasts, we could only compare the non-lethal *CRTAP* siblings to an age-matched healthy control as we had no bone samples from either lethal *CRTAP*-deficient individual for comparison.

Interestingly, it was recently reported that *TMEM38B* mutations caused an alteration in mitochondrial morphology in osteoblasts, with an elongated shape and evidence of cristolysis [44]. Our *CRTAP*-null individuals appear to have similar characteristics, with thinner mitochondria of increased length, suggesting increased mitochondrial fusion or failure to fission. Mitochondrial fusion occurs in response to cellular stress, such as starvation or inhibition of protein synthesis [45]. Gremminger et al. have also reported mitochondrial dysfunction in the *oim*/*oim* mouse, with reduced mitochondrial respiration in the muscles [46], suggesting that mitochondrial dysfunction may be a common alteration in multiple OI types. Additionally, inhibition of the mitochondrial DNA polymerase gamma (*Polg*) in osteoblasts demonstrated accelerated bone loss due to reduced bone formation and increased osteoclastogenesis in mice [47].

Transcripts regulating the expression of cell adhesion proteins were decreased throughout osteoblast differentiation in NL type VII OI *CRTAP*-null osteoblasts, although it remains unknown whether there is a corresponding decrease in adhesion proteins. We have previously shown that *CRTAP*-null fibroblasts deposit less type I collagen into the extracellular matrix [35], and have now demonstrated that steady-state collagen trafficking through the Golgi appears delayed following an ascorbic acid stimulation, leading to an increase in collagen retention in the ER. The paucity of overmodified collagen in the matrix together with a decrease in cellular adhesion proteins could alter the cellular cytoskeleton due to its decreased ability to bind to the extracellular matrix. In fact, lineage commitment of mesenchymal stem cells is influenced by actin remodeling, differentiating towards osteoblasts when there are higher levels of polymerization and towards adipocytes when actin is disrupted [48]. An abnormal cytoskeleton has been demonstrated in another dominant OI mouse model, the Brtl^+/−^ mouse, with increased disorganization of the cytoskeletal network leading to a more severe phenotype [49]. Decreased cellular adhesion proteins were also noted during differentiation of type XIV OI, including decreases in some of the cadherin-related pathways that we see here (*CELSR2*, *PCDHGA7*, *PCDHGA12*, *ICAM1*, and *TJP1*) [44], suggesting that it may be a common mechanism of multiple OI types.

Although human osteoblasts have not previously been examined, RNA-seq has been performed on murine *Crtap*^−/−^ long bone mixed cell populations enriched in osteocytes. Zimmerman et al. compared transcriptomics of RNA harvested from long bones of WT, *Crtap*^−/−^, and *oim*/*oim* mice [50]. While each OI mouse had many differentially expressed genes compared to WT (855 in *Crtap*^−/−^ and 544 in *oim*/*oim*), there were only 49 genes that were differently expressed from each other, suggesting common transcriptional pathways exist in the OI mouse models. Like *Crtap*^−/−^ mouse osteocytes, mature human type VII OI osteoblasts at day 21 of differentiation have significantly increased levels of *ALPL*, *IBSP*, and *DMP1* as well as *SMPD3*, *LOXL2*, and *IFITM5*. However, the genes most highly upregulated in the *Crtap*^−/−^ murine bone, including *Col1a1*, *Col1a2*, *Bglap*, and *Sparc*, were not upregulated in the human osteoblasts at any timepoints. Interestingly, there is also a significant decrease in both *COL1A1* and *COL1A2* in type VII *CRTAP*-null osteoblasts at day 14 of differentiation. Instead of increases in bone matrix proteins, we saw an increase in genes involved in cell division and replication. These cell cycle changes would not be seen in osteocytes as they are embedded in the bone matrix and are no longer proliferative. During osteoblast differentiation, proliferative ability slows as osteoblasts differentiate and mature.

The histological assessment of OI bones shows an increased amount of woven bone in more severe types of OI, which is thought to be deposited into tissue by less mature osteoblasts than for lamellar bone [51]. While the milder hypomorphic *CRTAP* c.[472-1021C > G]; [472-1021C > G] variant had histology similar to type I OI [2], histology from a perinatal lethal individual with a homozygous CRTAP p.(Ser135fs) variant showed the presence of woven bone [21]. Additionally, in a 33-year old patient with *CRTAP* c.[621 + 1G > A]; [1153-3C > G], bone histomorphometry showed a decreased osteoblast number and osteoid volume with little active bone formation [22]. In *Crtap*^−/−^ mice, histomorphometry showed a decreased bone volume per total volume (BV/TV), bone formation rate (BFR), mineralization lag time (Mlt), and osteoid surface [10]. Furthermore, the lack of CRTAP had an effect on tissues outside of the skeleton, such as the lungs and kidneys. Notably, these tissues were also shown to have increased proliferation upon BrdU staining [10].

Transforming growth factor β (TGF-β) and BMP-2, a TGF-β family member, are known to induce both osteoblast and chondrocyte differentiation as well as proliferation [52,53,54]. *Crtap*^−/−^ mice have an increased expression of TGF-β target genes *Cdkn1* and *Serpine1* in the calvarial bone, together with an increase in the proportion of phosphorylated SMAD2 to total SMAD2, suggesting that CRTAP deficiency elevates TGF-β signaling in these mice [55]. Grafe et al. hypothesized that the loss of collagen prolyl 3-hydroxylation by the complex leads to a decreased binding of collagen to decorin, which, when bound, can sequester TGF-β [55,56]. A decrease in BMP2 antagonists, *GREM1* and *GREM2*, together with an increase in *BMP2* expression in our probands, suggests that BMP2 likewise plays a role in the *CRTAP*-null osteoblast phenotype. As ossification and mineralization pathways are already increased in undifferentiated day 0 *CRTAP*-null osteoblasts (Figure 5a), they are primed to begin maturation, potentially from early signaling of TGF-β/BMP2-responsive genes such as *ALPL*, *MATN3*, and *MSX2*. As normal osteoblasts mature, they decrease in proliferation. The continued increase in proliferation transcripts through the differentiation timecourse despite increased *BMP2* pushing cells towards differentiation may suggest that although committed osteoblasts are differentiating faster, a population of pre-osteoblastic cells continues to proliferate when only mature cells should be present.

Since CRTAP was first identified as a cartilage protein expressed in hypertrophic chondrocytes [57] and CRTAP deficiency causes an osteochondrodysplasia [4], differences in both chondrocyte and osteoblasts would be expected. *MSX2* and *MATN3* transcripts are also elevated during chondrocyte differentiation along with *COL10A1*, which is normally present in hypertrophic chondrocytes, suggesting chondrocyte differentiation is enhanced as well (Supplementary Table S3) and that further investigation into chondrocytes would be warranted.

*P3H1* mutations, causing type VIII OI, are phenotypically similar to type VII OI, as their gene products mutually stabilize each other in vivo [9]. No detailed study of RNA expression in either human or mouse *P3H1*-null osteoblasts has been conducted. Although osteoblasts have not been studied, both types, VII [42] and VIII OI [58] bones, have a been examined by qBEI, with both having significant increases in CaPeak and CaHigh in cortical and cancellous bone compared to control. Although there were some differences in *P3H1*-null bone, such as increased levels of lowly mineralized collagen (CaLow) and the presence of patchy osteoid [58], the common lack of both CRTAP and P3H1 protein in type VIII OI individuals may suggest there are similarities in disease mechanism. It is tempting to speculate that CRTAP, although it lacks enzyme activity, is the more important partner in maintaining the 3-hydroxylation complex, as patients without CRTAP lack P3H1 protein, but individuals with null *P3H1* mutations, still have a residual amount of CRTAP [9] and non-lethal type VIII OI is much more frequent than non-lethal type VII.

In conclusion, we have shown that the *CRTAP*-null osteoblast transcriptional phenotype is multi-faceted, with increased proliferation and increased cellular differentiation. While increased proliferation provides a larger population of immature cells, if not properly differentiated, they will not deposit the lamellar bone of mature cells needed for both strength and integrity. The dysregulation of osteoblast differentiation, together with changes in cellular adhesion may contribute to the extreme severity of the *CRTAP*-null phenotype.

## Figures and Tables

**Table 1:** Hydroxyproline and hydroxylysine analysis of secreted collagen.

Samples	Hyp% (FB)	Hyl% (FB)	P986 3-OH (FB)	P986 3-OH (OB)
Controls	42.7–49.4	22.5–33.8	95–98	95
NL-1	46.1	48.1	0	0
NL-2	44.5	49.1	4	ND
L-1	45.8	48.4	0 *	ND
L-2	44.7	42.9	0 **	ND

Controls: n = 3 (FB) or 1 (OB); * infant 2 [3]; ** proband 4 [9]; Hyp%: (hydroxyproline/(proline + hydroxyproline)) × 100; Hyl%: (hydroxylysine/(lysine + hydroxylysine)) × 100; FB = fibroblasts; OB = osteoblasts.

**Table 2:** Upregulated gene ontology (GO) secretion pathways.

GO Pathway	Description	*p*-adj	Pathway Genes
GO: 0051047	positive regulation of secretion	0.000937	FGR, PTPN22, AIM2, AGT, INHBB, CACNA1D, TRH, SNCA, NPY2R, C1QTNF3, F2R, F2RL1, TSLP, HLA-F, AQP1, ZP3, BLK, CCL19, GATA3, CASP1, IGF1, P2RX7, RPH3AL, ALOX15B, CACNA1G, MYOM1, MBP, NLRP2, OPRL1, ITGB2, APLN
GO: 1903532	positive regulation of secretion by cell	0.00132	FGR, PTPN22, AIM2, AGT, INHBB, CACNA1D, TRH, SNCA, NPY2R, C1QTNF3, F2R, F2RL1, TSLP, HLA-F, ZP3, BLK, CCL19, GATA3, CASP1, IGF1, P2RX7, RPH3AL, ALOX15B, CACNA1G, MYOM1, MBP, NLRP2, ITGB2, APLN
GO: 0050715	positive regulation of cytokine secretion	0.00581	FGR, PTPN22, AIM2, C1QTNF3, F2R, F2RL1, TSLP, CCL19, GATA3, CASP1, P2RX7, ALOX15B, MBP, NLRP2
GO: 0002791	regulation of peptide secretion	0.00581	FGR, PTPN22, AIM2, INHBB, CACNA1D, TRH, NPY2R, C1QTNF3, F2R, F2RL1, TSLP, CD74, BLK, CHD7, CCL19, GATA3, ADRA2A, CASP1, CARD16, IGF1, P2RX7, RPH3AL, ALOX15B, NR1D1, MYOM1, MBP, C5AR2, NLRP2, CD40, APLN
GO: 0002793	positive regulation of peptide secretion	0.00581	FGR, PTPN22, AIM2, TRH, NPY2R, C1QTNF3, F2R, F2RL1, TSLP, BLK, CCL19, GATA3, CASP1, IGF1, P2RX7, RPH3AL, ALOX15B, MYOM1, MBP, NLRP2, APLN
GO: 0050663	cytokine secretion	0.0096	FGR, GBP5, PTPN22, AIM2, AGT, C1QTNF3, F2R, F2RL1, TSLP, CCL19, GATA3, CASP1, CARD16, P2RX7, ALOX15B, MBP, C5AR2
GO: 0050702	interleukin-1 beta secretion	0.01	GBP5, AIM2, F2RL1, CCL19, CASP1, CARD16, P2RX7, NLRP2
GO: 0050714	positive regulation of protein secretion	0.0108	FGR, PTPN22, AIM2, TRH, C1QTNF3, F2R, F2RL1, TSLP, BLK, CCL19, GATA3, CASP1, IGF1, P2RX7, RPH3AL, ALOX15B, MYOM1, MBP, NLRP2
GO: 0050707	regulation of cytokine secretion	0.0138	FGR, PTPN22, AIM2, C1QTNF3, F2R, F2RL1, TSLP, CCL19, GATA3, CASP1, CARD16, P2RX7, ALOX15B, MBP, C5AR2, NLRP2
GO: 0050701	interleukin-1 secretion	0.018	GBP5, AIM2, F2RL1, CCL19, CASP1, CARD16, P2RX7, NLRP2
GO: 0050708	regulation of protein secretion	0.0229	FGR, PTPN22, AIM2, INHBB, CACNA1D, TRH, C1QTNF3, F2R, F2RL1, TSLP, BLK, CCL19, GATA3, ADRA2A, CASP1, CARD16, IGF1, P2RX7, RPH3AL, ALOX15B, NR1D1, MYOM1, MBP, C5AR2, NLRP2, CD40

## Data Availability

The RNA-sequencing dataset produced in this study is deposited at dbGaP under the accession code phs003969.v1.p1. To access these data, users may apply for access to the dbGaP data repository (http://www.ncbi.nlm.nih.gov/projects/gap/cgi-bin/study.cgi?study_id=phs003969.v1.p1 (accessed on 30 January 2025)).

## Supplementary Materials

The following supporting information can be downloaded at: https://www.mdpi.com/article/10.3390/cells14070518/s1, Supplementary information: Extended clinical report on the *CRTAP*-null individuals, Supplementary Table S1: qPCR Taqman Assays, Supplementary Table S2: Differentially expressed genes during osteoblast differentiation between control and *CRTAP*-null osteoblasts, Supplementary Table S3: Upregulated chondrocyte GO pathways during *CRTAP*-null osteoblast differentiation; Supplementary Table S4: Downregulated chondrocyte GO pathways during *CRTAP*-null osteoblast differentiation. References [33,34,59] are cited in Supplementary Materials.

## References

[1] Forlino A., Marini J.C. (2016). Osteogenesis imperfecta. Lancet.

[2] Ward L.M., Rauch F., Travers R., Chabot G., Azouz E.M., Lalic L., Roughley P.J., Glorieux F.H. (2002). Osteogenesis imperfecta type VII: An autosomal recessive form of brittle bone disease. Bone.

[3] Barnes A.M., Chang W., Morello R., Cabral W.A., Weis M., Eyre D.R., Leikin S., Makareeva E., Kuznetsova N., Uveges T.E. (2006). Deficiency of cartilage-associated protein in recessive lethal osteogenesis imperfecta. N. Engl. J. Med..

[4] Morello R., Bertin T.K., Chen Y., Hicks J., Tonachini L., Monticone M., Castagnola P., Rauch F., Glorieux F.H., Vranka J. (2006). CRTAP is required for prolyl 3-hydroxylation and mutations cause recessive osteogenesis imperfecta. Cell.

[5] Marini J.C., Cabral W.A., Barnes A.M., Chang W. (2007). Components of the collagen prolyl 3-hydroxylation complex are crucial for normal bone development. Cell Cycle.

[6] Cabral W.A., Chang W., Barnes A.M., Weis M., Scott M.A., Leikin S., Makareeva E., Kuznetsova N.V., Rosenbaum K.N., Tifft C.J. (2007). Prolyl 3-hydroxylase 1 deficiency causes a recessive metabolic bone disorder resembling lethal/severe osteogenesis imperfecta. Nat Genet..

[7] Barnes A.M., Carter E.M., Cabral W.A., Weis M., Chang W., Makareeva E., Leikin S., Rotimi C.N., Eyre D.R., Raggio C.L. (2010). Lack of cyclophilin B in osteogenesis imperfecta with normal collagen folding. N. Engl. J. Med..

[8] Ishikawa Y., Wirz J., Vranka J.A., Nagata K., Bächinger H.P. (2009). Biochemical characterization of the prolyl 3-hydroxylase 1.cartilage-associated protein.cyclophilin B complex. J. Biol. Chem..

[9] Chang W., Barnes A.M., Cabral W.A., Bodurtha J.N., Marini J.C. (2010). Prolyl 3-hydroxylase 1 and CRTAP are mutually stabilizing in the endoplasmic reticulum collagen prolyl 3-hydroxylation complex. Hum. Mol. Genet..

[10] Baldridge D., Lennington J., Weis M., Homan E.P., Jiang M.M., Munivez E., Keene D.R., Hogue W.R., Pyott S., Byers P.H. (2010). Generalized connective tissue disease in Crtap-/- mouse. PLoS ONE.

[11] Grol M.W., Haelterman N.A., Lim J., Munivez E.M., Archer M., Hudson D.M., Tufa S.F., Keene D.R., Lei K., Park D. (2021). Tendon and motor phenotypes in the Crtap(-/-) mouse model of recessive osteogenesis imperfecta. eLife.

[12] Alanay Y., Avaygan H., Camacho N., Utine G.E., Boduroglu K., Aktas D., Alikasifoglu M., Tuncbilek E., Orhan D., Bakar F.T. (2010). Mutations in the gene encoding the RER protein FKBP65 cause autosomal-recessive osteogenesis imperfecta. Am. J. Hum. Genet..

[13] Amor I.M., Rauch F., Gruenwald K., Weis M., Eyre D.R., Roughley P., Glorieux F.H., Morello R. (2011). Severe osteogenesis imperfecta caused by a small in-frame deletion in CRTAP. Am. J. Med. Genet. A.

[14] Baldridge D., Schwarze U., Morello R., Lennington J., Bertin T.K., Pace J.M., Pepin M.G., Weis M., Eyre D.R., Walsh J. (2008). CRTAP and LEPRE1 mutations in recessive osteogenesis imperfecta. Hum. Mutat..

[15] Holtz A.P., Souza L.T., Ribeiro E.M., Acosta A.X., Lago R.M., Simoni G., Llerena J.C., Félix T.M. (2023). Genetic analysis of osteogenesis imperfecta in a large Brazilian cohort. Bone.

[16] Kuptanon C., Thamkunanon V., Srichomthong C., Theerapanon T., Suphapeetiporn K., Porntaveetus T., Shotelersuk V. (2022). Novel BMP1, CRTAP, and SERPINF1 variants causing autosomal recessive osteogenesis imperfecta. Clin. Genet..

[17] Madhuri V., Selina A., Loganathan L., Kumar A., Kumar V., Raymond R., Ramesh S., Vincy N., Joel G., James D. (2021). Osteogenesis imperfecta: Novel genetic variants and clinical observations from a clinical exome study of 54 Indian patients. Ann. Hum. Genet..

[18] Marulanda J., Ludwig K., Glorieux F., Lee B., Sutton V.R., Retrouvey J.M., Rauch F., Members of the BBD Consortium (2022). Craniofacial and dental phenotype of two girls with osteogenesis imperfecta due to mutations in CRTAP. Bone.

[19] Tang Y.A., Wang L.Y., Chang C.M., Lee I.W., Tsai W.H., Sun H.S. (2020). Novel Compound Heterozygous Mutations in CRTAP Cause Rare Autosomal Recessive Osteogenesis Imperfecta. Front. Genet..

[20] Tuysuz B., Elkanova L., Alkaya D.U., Güleç Ç., Toksoy G., Güneş N., Yazan H., Bayhan A.I., Yıldırım T., Yeşil G. (2022). Osteogenesis imperfecta in 140 Turkish families: Molecular spectrum and, comparison of long-term clinical outcome of those with COL1A1/A2 and biallelic variants. Bone.

[21] Van Dijk F.S., Nesbitt I.M., Nikkels P.G., Dalton A., Bongers E.M., Van De Kamp J.M., Hilhorst-Hofstee Y., Den Hollander N.S., Lachmeijer A., Marcelis C.L. (2009). CRTAP mutations in lethal and severe osteogenesis imperfecta: The importance of combining biochemical and molecular genetic analysis. Eur. J. Hum. Genet..

[22] Zhou B., Gao P., Hu J., Lin X., Sun L., Zhang Q., Jiang Y., Wang O., Xia W., Xing X. (2024). Genetic analysis, phenotype spectrum and functional study of rare osteogenesis imperfecta caused by CRTAP Variants. J. Clin. Endocrinol. Metab..

[23] Robey P.G., Termine J.D. (1985). Human bone cells in vitro. Calcif. Tissue Int..

[24] Bonadio J., Byers P.H. (1985). Subtle structural alterations in the chains of type I procollagen produce osteogenesis imperfecta type II. Nature.

[25] Nagashima K., Zheng J., Parmiter D., Patri A.K. (2011). Biological tissue and cell culture specimen preparation for TEM nanoparticle characterization. Methods in Molecular Biology.

[26] Seref-Ferlengez Z., Kennedy O.D., Schaffler M.B. (2015). Bone microdamage, remodeling and bone fragility: How much damage is too much damage?. Bonekey Rep..

[27] Andrews S. (2010). FastQC: A Quality Control Tool for High Throughput Sequence Data. http://www.bioinformatics.babraham.ac.uk/projects/fastqc/.

[28] Martin M. (2011). Cutadapt removes adapter sequences from high-throughput sequencing reads. EMBnet. J..

[29] Dobin A., Davis C.A., Schlesinger F., Drenkow J., Zaleski C., Jha S., Batut P., Chaisson M., Gingeras T.R. (2013). STAR: Ultrafast universal RNA-seq aligner. Bioinformatics.

[30] Liao Y., Smyth G.K., Shi W. (2014). featureCounts: An efficient general purpose program for assigning sequence reads to genomic features. Bioinformatics.

[31] Love M.I., Huber W., Anders S. (2014). Moderated estimation of fold change and dispersion for RNA-seq data with DESeq2. Genome Biol..

[32] Yu G., Wang L.G., Han Y., He Q.Y. (2012). clusterProfiler: An R package for comparing biological themes among gene clusters. OMICS A J. Integr. Biol..

[33] Shaheen R., Alazami A.M., Alshammari M.J., Faqeih E., Alhashmi N., Mousa N., Alsinani A., Ansari S., Alzahrani F., Al-Owain M. (2012). Study of autosomal recessive osteogenesis imperfecta in Arabia reveals a novel locus defined by TMEM38B mutation. J. Med. Genet..

[34] Gochuico B.R., Hossain M., Talvacchio S.K., Zuo M.X., Barton M., Do A.N., Marini J.C. (2023). Pulmonary function and structure abnormalities in children and young adults with osteogenesis imperfecta point to intrinsic and extrinsic lung abnormalities. J. Med. Genet..

[35] Valli M., Barnes A.M., Gallanti A., Cabral W.A., Viglio S., Weis M.A., Makareeva E., Eyre D., Leikin S., Antoniazzi F. (2012). Deficiency of CRTAP in non-lethal recessive osteogenesis imperfecta reduces collagen deposition into matrix. Clin. Genet..

[36] Foulkes W.D., Flanders T.Y., Pollock P.M., Hayward N.K. (1997). The CDKN2A (p16) gene and human cancer. Mol. Med..

[37] Pines J., Hunter T. (1989). Isolation of a human cyclin cDNA: Evidence for cyclin mRNA and protein regulation in the cell cycle and for interaction with p34cdc2. Cell.

[38] Amano K., Ichida F., Sugita A., Hata K., Wada M., Takigawa Y., Nakanishi M., Kogo M., Nishimura R., Yoneda T. (2008). MSX2 stimulates chondrocyte maturation by controlling Ihh expression. J. Biol. Chem..

[39] Muttigi M.S., Han I., Park H.K., Park H., Lee S.H. (2016). Matrilin-3 Role in Cartilage Development and Osteoarthritis. Int. J. Mol. Sci..

[40] Yang X., Trehan S.K., Guan Y., Sun C., Moore D.C., Jayasuriya C.T., Chen Q. (2014). Matrilin-3 inhibits chondrocyte hypertrophy as a bone morphogenetic protein-2 antagonist. J. Biol. Chem..

[41] Cambier S., Gline S., Mu D., Collins R., Araya J., Dolganov G., Einheber S., Boudreau N., Nishimura S.L. (2005). Integrin alpha(v)beta8-mediated activation of transforming growth factor-beta by perivascular astrocytes: An angiogenic control switch. Am. J. Pathol..

[42] Fratzl-Zelman N., Morello R., Lee B., Rauch F., Glorieux F.H., Misof B.M., Klaushofer K., Roschger P. (2010). CRTAP deficiency leads to abnormally high bone matrix mineralization in a murine model and in children with osteogenesis imperfecta type VII. Bone.

[43] Arslan M.A., Chikina M., Csermely P., Sőti C. (2012). Misfolded proteins inhibit proliferation and promote stress-induced death in SV40-transformed mammalian cells. FASEB J..

[44] Jovanovic M., Mitra A., Besio R., Contento B.M., Wong K.W., Derkyi A., To M., Forlino A., Dale R.K., Marini J.C. (2023). Absence of TRIC-B from type XIV Osteogenesis Imperfecta osteoblasts alters cell adhesion and mitochondrial function—A multi-omics study. Matrix Biol..

[45] Adebayo M., Singh S., Singh A.P., Dasgupta S. (2021). Mitochondrial fusion and fission: The fine-tune balance for cellular homeostasis. FASEB J..

[46] Gremminger V.L., Jeong Y., Cunningham R.P., Meers G.M., Rector R.S., Phillips C.L. (2019). Compromised Exercise Capacity and Mitochondrial Dysfunction in the Osteogenesis Imperfecta Murine (oim) Mouse Model. J. Bone Miner. Res..

[47] Dobson P.F., Dennis E.P., Hipps D., Reeve A., Laude A., Bradshaw C., Stamp C., Smith A., Deehan D.J., Turnbull D.M. (2020). Mitochondrial dysfunction impairs osteogenesis, increases osteoclast activity, and accelerates age related bone loss. Sci. Rep..

[48] Khan A.U., Qu R., Fan T., Ouyang J., Dai J. (2020). A glance on the role of actin in osteogenic and adipogenic differentiation of mesenchymal stem cells. Stem Cell Res. Ther..

[49] Bianchi L., Gagliardi A., Maruelli S., Besio R., Landi C., Gioia R., Kozloff K.M., Khoury B.M., Coucke P.J., Symoens S. (2015). Altered cytoskeletal organization characterized lethal but not surviving Brtl+/− mice: Insight on phenotypic variability in osteogenesis imperfecta. Hum. Mol. Genet..

[50] Zimmerman S.M., Dimori M., Heard Lipsmeyer M.E., Morello R. (2019). The Osteocyte Transcriptome Is Extensively Dysregulated in Mouse Models of Osteogenesis Imperfecta. J. Bone Miner. Res. Plus.

[51] Shapiro F., Maguire K., Swami S., Zhu H., Flynn E., Wang J., Wu J.Y. (2021). Histopathology of osteogenesis imperfecta bone. Supramolecular assessment of cells and matrices in the context of woven and lamellar bone formation using light polarization and ultrastructural microscopy. Bone Rep..

[52] Chen G., Deng C., Li Y.P. (2012). TGF-beta and BMP signaling in osteoblast differentiation and bone formation. Int. J. Biol. Sci..

[53] Beederman M., Lamplot J.D., Nan G., Wang J., Liu X., Yin L., Li R., Shui W., Zhang H., Kim S.H. (2013). BMP signaling in mesenchymal stem cell differentiation and bone formation. J. Biomed. Sci. Eng..

[54] Shu B., Zhang M., Xie R., Wang M., Jin H., Hou W., Tang D., Harris S.E., Mishina Y., O’Keefe R.J. (2011). BMP2, but not BMP4, is crucial for chondrocyte proliferation and maturation during endochondral bone development. J. Cell Sci..

[55] Grafe I., Yang T., Alexander S., Homan E.P., Lietman C., Jiang M.M., Bertin T., Munivez E., Chen Y., Dawson B. (2014). Excessive transforming growth factor-beta signaling is a common mechanism in osteogenesis imperfecta. Nat. Med..

[56] Markmann A., Hausser H., Schönherr E., Kresse H. (2000). Influence of decorin expression on transforming growth factor-beta-mediated collagen gel retraction and biglycan induction. Matrix Biol..

[57] Castagnola P., Gennari M., Morello R., Tonachini L., Marin O., Gaggero A., Cancedda R. (1997). Cartilage associated protein (CASP) is a novel developmentally regulated chick embryo protein. J. Cell Sci..

[58] Fratzl-Zelman N., Barnes A.M., Weis M., Carter E., Hefferan T.E., Perino G., Chang W., Smith P.A., Roschger P., Klaushofer K. (2016). Non-Lethal Type VIII Osteogenesis Imperfecta Has Elevated Bone Matrix Mineralization. J. Clin. Endocrinol. Metab..

[59] Hall J.G., Gripp K.W., Slavotinek A.M. (2006). Hall’s Handbook of Physical Measurements.

