# Supplementary material for: CRTAP-Null Osteoblasts Have Increased Proliferation, Protein Secretion, and Skeletal Morphogenesis Gene Expression with Downregulation of Cellular Adhesion
Source: Cells. 2025 Mar 31;14(7):518. doi: 10.3390/cells14070518 (PMC11988066; doi:10.3390/cells14070518)
Supplement: Supplementary file 1 [file cells-14-00518-s001.zip › Supplementary clinical information.docx]

**Supplementary information:** Extended clinical report on the *CRTAP*-null individuals

*Clinical report*

**Proband 1 (NL-1)** is an 8-year-old girl at the time of this report with Type VII OI, whose parents come from Saudi Arabia and are first cousins. She is the fifth child of 5 live births with 2 older healthy siblings, an older sibling with OI type VII (**NL-2**), and a sibling deceased at 2 months of age (**Figure 1a, II.5**). She was born in the United States at term to a G5P4 mother via cesarean section for malpresentation. At birth, she weighed 2.77 kg (< 3^rd^ centile) and had 18 fractures. She was intubated for 5 days and received intensive care for 3 weeks after birth. She was confirmed to be homozygous for the *CRTAP* variant p.[(Tyr187*)] ; [(Tyr187*)](**Figure 1b**).

This proband was 2 years 4 months old at enrollment in the NICHD, NIH study. At her initial evaluations, her measurements were length 59 cm (< 3^rd^, 50^th^ centile for 3-month-old), weight 6.5 kg (< 3^rd^, 50^th^ centile for 4.5-month-old), head circumference 45.5 cm (35^th^ centile). She was noted to have brachycephaly, triangular facies, flat midface, low set ears and blue scleral hue. Dentinogenesis imperfecta was not present, although there was delayed dental eruption (possibly due to extensive prior treatment with bisphosphonates) and poor spacing of primary teeth. On physical exam, her lungs were clear to auscultation, cardiac exam was normal, and a lower thoracic kyphosis was noted. Limb evaluations showed hyper flexibility in lower extremities, bowing of L humerus, L radius, and bilateral tibia and fibula. Pes planus was noted; there were no contractures. Gross motor and intellectual development were delayed. At her initial evaluation, she could feed herself with her hands or a spoon. She had good head control and was able to sit with support. She propelled herself by scooting on her back or from a sitting position for distances of 6-8 feet and was non-weight bearing. She had delayed speech development and limited social interactions. Radiographs at the initial visit showed 12^th^ rib agenesis, diffuse osteopenia with multiple thoracic and lumbar vertebral compressions, most prominently at L1/L2 and levoscoliosis (**Figure 1d-e**).

During the first 2 years of life, she experienced multiple fractures of long bones, including at least 3 femur fractures, as well as ribs and vertebral. After her second birthday, she underwent her first femoral surgical rodding with closed osteoclasis (**Figure 1c**). Two years later, her bilateral femur rods were replaced with expandable rods. She subsequently had additional long bone fractures that were managed with casting. Between 2019 and 2024, she had five long bone surgeries with rod replacements in both upper and lower limbs.

She developed moderate scoliosis, and “popcorn” deformities at the distal femoral and proximal tibial metaphyses bilaterally. At 3 years of age, her L1-L4 DXA z-score was -7.3, with a raw bone mineral density of 0.218 g/cm^2^. She continued to have DXA scans yearly, with DXA scores of – 5.8, -4.8 and -6.3 at ages 4, 6, and 7 years, respectively.

Her growth continued to be severely impaired, with length progressing between the 50-75^th^ centile curve for individuals with OI type III OI (**Figure 1f).** At age 4 years 5 months, her arm span was 85 cm and her upper segment/lower segment (US/LS) ratio was 1.74 (> newborn)[1]. The mean US/LS ratio at 4 years 5 months is 1.18. Her current height of 83 cm is 50^th^ centile for 21-month-old girl without OI.

She underwent a multi-disciplinary evaluation at 3 years 11 months of age. Neuropsychometric evaluation noted delays in receptive/expressive language, as well as gross and fine motor skills. All areas of adaptive functioning were delayed, with her adaptive motor skills showing the most severe delay. She pulled to standing after rodding surgery at age 5 years 1 month. At 8 years of age, she can sit unsupported, has good head control and can stand with an object for support. She understands both English and Arabic.

Examination of relevant physical systems showed:

**Audiology:** Normal bilateral ear mobility with slightly negative middle ear

pressure. By 6 years 5 months of age, mild conductive hearing loss was detected bilaterally at 250 Hz in the right ear and 250-500 Hz in the left ear.

**Ophthalmology:** At 5 years 10 months of age, intermittent esotropia, early signs of amblyopia and significantly thin cornea were noted.

**Cranial Exam:** At 3 years 11 months, head CT showed brachycephaly, Wormian bones involving the lambdoid sutures, and platybasia without basilar invagination. Her head CT remained stable.

**CardioPulmonary:** Her echocardiogram and EKG were normal.

On chest CT, she has scarring in the right upper lung which is stable.

Pulmonary function tests were normal with no obstruction or restriction

detected on spirometry. There was no history of significant respiratory

illnesses, such as pneumonia.

**Bone Metabolism**: Most bone-related laboratory values (IGF, ALP, BSALP,

Vitamin D), were within reference ranges, except for elevated osteocalcin.

Her highest osteocalcin value was 58.4 ng/mL at age 3 years 11 months (reference range 7.3-38.5 ng/mL). Serum osteocalcin decreased to 48.1 ng/mL at 6 years 5 months of age.

**Developmental:** Due to developmental concerns, the patient underwent an extensive developmental evaluation. All areas of adaptive functioning were delayed, with her adaptive motor skills showing the most severe delay. She pulled to standing after rodding surgery at age 5 years 1 month. At 8 years of age, she can sit unsupported, has good head control and can stand with an object for support. She understands both English and Arabic.

**Pharmacology:** She began to receive 1 mg/kg pamidronate infusions on day 3 of life and continues the infusions to the present at Children’s National Hospital.

**Proband 2 (NL-2)** is a 14 years 2 months old girl at the time of this report with type VII OI. She is the older sister of Proband 1. She was born in Saudi Arabia, where she was initially identified as having type VII OI [2]. She was the product of a full-term gestation to a G4P3 mother, delivered via cesarean section secondary to findings of 15-18 fractures on pre-natal ultrasound (**Figure 1a, II.4**). At birth, she weighed 2.29 kg (< 3^rd^ centile) and had fractures in the left clavicle, left femur, and right tibia/fibula. She was admitted to the NICU after birth for 5 days. Genetic testing identified a homozygous NM_006371.4: c.[561T>G] ; NP_006362.1: p.[(Tyr187*)] variant in *CRTAP*. Her parents emigrated to the US soon after her birth for further care.

The proband was 8 years 3 months old when she enrolled in the OI program at NICHD, NIH. At her initial evaluations, her measurements were length 85.7 cm (<3^rd^, 50^th^ percentile for 2 years 3 months old), weight 20.1 kg (< 3^rd^, 50^th^ percentile for 6 year old), head circumference 52 cm (50-75^th^ percentile). Between 6 years to 13 years 6 months her length remained below 3^rd^ centile on the standard growth curve and 50-75^th^ centile on the OI Type III curve (**Figure 1f**). The proband was noted to have triangular face, flat mid-face, low set ears and light blue scleral hue. Dentinogenesis imperfecta was not present although a late eruption pattern was noted. She had refractive amblyopia requiring corrective lens and mild hyperopic astigmatism. Her hearing was normal. She had a barrel chest and mild kyphoscoliosis. Limb physical findings included bowing of the radius bilaterally, contractures at the elbows, hip laxity (able to touch her feet to her head), and pes planus. Her arm span at age 12 years 7 months was 127 cm, and her US/LS ratio was 1.39 (average for a 2.2 year old girl)[1]. The mean US/LS ratio for females at 12 years 7 months is 1. Her motor development was delayed. She had achieved head control and scooting on her back by one year of age, sat unsupported at 2 years, and stood with a gait trainer at 8 years of age. She never achieved an ability to crawl, pull to stand, or stand independently. At 13 years 6 months of age, she uses an electric wheelchair for mobility and can transfer independently from and to the chair. She has age-appropriate cognitive and social development, able to communicate in both English and Arabic and excels in school.

During the first year of life, she sustained 15-20 long bone fractures, including at least 4 femur fractures, with movements such as scooting; fractures were managed by splinting. The proband had bilateral femoral and tibial rods placed at ~2 years and humeral and radial rods at ~3 years of age (**Figure 1g-h**). She continued to have multiple long bone fractures yearly and underwent lower limb rod revisions at age 4 and 5 years, and upper limb rod revision at age 6 years. At 9 years, she sustained upper limb and left tibial fractures following falling out of parent’s arms during transfer, requiring rod revision. Fear of refracturing led to decreased independent activities and use of her gait trainer. Between 2012 and 2023 she has had ten surgeries and more than twenty-five fractures. Skeletal radiographs showed undertubulated long bone shafts and diffused osteopenia with loss of vertebral body height throughout the thoracolumbar spine. She has moderately increasing scoliosis in her lower thorax, minimal wedging in her upper lumbar vertebrae, anterior wedging in her lower thorax, osteopenia, and loss of vertebral height throughout the thoracolumbar spine (**Figure 1i-j**). Her L1-L4 DXA z-score was -4.17 at 6 years and ranged from -2.9 (at 9 years 3 months) to 0 (at 11 years 4 months; bone mineral density 0.42 – 0.69 g/cm^2^), and at 13 years 6 months is z = -2.1.

Results of evaluations of relevant physical systems include:

**Dental**: At 12 years 7 months of age, delayed eruption and bilateral posterior open bite and crossbite with mild discoloration were noted.

**Audiology:** She has mild bilateral conductive hearing loss and a hypercompliant middle ear system in the left ear which is stable.

**Cranial exam:** Head CT showed lamboidal Wormian bones without platybasia or basilar invagination, and an enlarged cerebrospinal fluid space without hydrocephalus.

**CardioPulmonary:** Mildly dilated ascending aorta and bicuspid aortic valve were present on echocardiogram. The proband has a history of recurrent pneumonia at age 2-3 years and chronic lung disease secondary to recurrent aspiration and reflux. This resulted in a gastrotomy tube placement for supplemental nutrition until age 7. Chest CT showed right upper lobe scarring and non-specific sub-centimeter ground-glass nodules as

described[3]. Pulmonary function tests showed moderate to severe restrictive defect and mild lower airway obstruction. She developed symptoms of obstructive sleep apnea at ~9 years from decreased mobility and weight gain, consequences of being fearful of refracturing.

**Bone Metabolism:** Most bone-related laboratory values (IGF, ALP, BSALP) were within the reference ranges, except for an elevated osteocalcin of 51.8 -52.5 ng/mL (reference range 7.3-38.5 ng/mL) at ages 8 years 3 months and 13 years 6 months, respectively, and 25-OH vitamin D level ranging from 18-24 ng/mL.

**Pharmacology:** She was started on 1 mg/kg IV pamidronate on Day 20 of life and has continued this medication every 3 months at Children’s National Hospital in Washington D.C. since establishing care in the US.

References

1. Hall JG, A.J., Gripp KW, Slavotinek AM, *Hall’s Handbook of Physical Measurements*. 2006, Oxford: Oxford University Press.

2. Shaheen, R., et al., *Study of autosomal recessive osteogenesis imperfecta in Arabia reveals a novel locus defined by TMEM38B mutation.* J Med Genet, 2012. **49**(10): p. 630-5.

3. Gochuico, B.R., et al., *Pulmonary function and structure abnormalities in children and young adults with osteogenesis imperfecta point to intrinsic and extrinsic lung abnormalities.* J Med Genet, 2023. **60**(11): p. 1067-1075.
