# Supplementary material for: CRTAP-Null Osteoblasts Have Increased Proliferation, Protein Secretion, and Skeletal Morphogenesis Gene Expression with Downregulation of Cellular Adhesion
Source: Cells. 2025 Mar 31;14(7):518. doi: 10.3390/cells14070518 (PMC11988066; doi:10.3390/cells14070518)
Supplement: Supplementary file 1 [file cells-14-00518-s001.zip › Supplementary Table S1 - qPCR Taqman Assays.docx]

| *CRTAP* | Hs01035151_m1 |
| --- | --- |
| *P3H1* | Hs00223565_m1 |
| *PPIB* | Hs00168719_m1 |
| *COL1A1* | Hs00164004_m1 |
| *PLOD1* | Hs00609368_m1 |
| *ALPL* | Hs01029144_m1 |
| *IBSP* | Hs00173720_m1 |
| *MEPE* | Hs00220237_m1 |
| *SP7* | Hs01866874_s1 |
| *CCNB1* | Hs01030099_m1 |
| *CDKN2A* | Hs00923894_m1 |
| *BMP2* | Hs00154192_m1 |
| *MSX2* | Hs00751239_s1 |
| *MATN3* | Hs01111974_m1 |
| *GREM1* | Hs01879841_s1 |
| *GREM2* | Hs03986140_s1 |
| *TEK* | Hs00945150_m1 |
| *ITGB8* | Hs00174456_m1 |
| *CELSR2* | Hs00154903_m1 |
| *PCDHGA7* | Hs00259352_s1 |
| *PCDHGA12* | Hs00259391_s1 |
| *MMP24* | Hs00198580_m1 |
| *ICAM1* | Hs00164932_m1 |
| *ADAMTSL4* | Hs01120103_g1 |
| *ACAN* | Hs00153936_m1 |
| *TJP1* | Hs00543811_g1 |
| *GAPDH* | Hs02758991_g1 |
| *ACTB* | Hs99999903_m1 |
| *B2M* | Hs00984230_m1 |

Supplementary Table S1 – qPCR Taqman Assays
