# Supplementary material for: CRTAP-Null Osteoblasts Have Increased Proliferation, Protein Secretion, and Skeletal Morphogenesis Gene Expression with Downregulation of Cellular Adhesion
Source: Cells. 2025 Mar 31;14(7):518. doi: 10.3390/cells14070518 (PMC11988066; doi:10.3390/cells14070518)
Supplement: Supplementary file 1 [file cells-14-00518-s001.zip › Supplementary Table S3 Upregulated Chondrocyte genes.docx]

| **GO Pathway** | **Description** | ***p*-value** | ***p*-adjust** | **Gene ID** |
| --- | --- | --- | --- | --- |
| **UP - DAY 0** |  |  |  |  |
| GO:0002062 | chondrocyte differentiation | 1E-06 | 3.97E-05 | WNT2B, MATN3, VIT, RARB, PTH1R, POC1A, SHOX2, FGFR3, CYTL1, SFRP2, MSX2, BMP6, SCARA3, MATN2, CCN3, COL27A1, RFLNA, ZNF664-RFLNA, ACAN, BMP2 |
| GO:0002063 | chondrocyte development | 1.5E-06 | 5.35E-05 | MATN3, VIT, POC1A, SHOX2, SFRP2, MSX2, SCARA3, MATN2, COL27A1, RFLNA, ZNF664-RFLNA, ACAN |
| GO:0003418 | growth plate cartilage chondrocyte differentiation | 0.00066 | 0.0122 | MATN3, VIT, POC1A, SCARA3, MATN2, COL27A1 |
| GO:0003433 | chondrocyte development involved in endochondral b | 0.00066 | 0.0122 | MATN3, VIT, POC1A, SCARA3, MATN2, COL27A1 |
| GO:0003413 | chondrocyte differentiation involved in endochondrogenesis | 0.0023 | 0.0336 | MATN3, VIT, POC1A, SCARA3, MATN2, COL27A1 |
| **UP - DAY 7** |  |  |  |  |
| GO:0002062 | chondrocyte differentiation | 7.6E-05 | 0.0108 | VWA1, MATN3, VIT, RARB, PTH1R, FGFR3, CYTL1, TGFBI, MSX2, RFLNA, ZNF664-RFLNA, BMP2 |
| **UP - DAY 14** |  |  |  |  |
| GO:0002062 | chondrocyte differentiation | 0.00039 | 0.0122 | VWA1, VIT, RARB, PTH1R, SHOX2, FGFR3, CYTL1, SFRP2, TGFBI MSX2, SNAI2, RFLNA, ZNF664-RFLNA, |
| GO:0002063 | chondrocyte development | 0.00135 | 0.0303 | VWA1, VIT, SHOX2, SFRP2, MSX2, RFLNA, ZNF664-RFLNA |
| **UP - DAY 21** |  |  |  |  |
| GO:0002062 | chondrocyte differentiation | 9.21E-08 | 6.34E-06 | VWA1, WNT9A, VIT, RARB, PTH1R, POC1A, SHOX2,  FGFR3, CYTL1, NKX3-2, SFRP2, TGFBI, MSX2, LOXL2,  SCARA3, WNT10B, HMGA2, RFLNA, ZNF664-RFLNA,  SMPD3, BMP2, MATN4 |
| GO:0002063 | chondrocyte development | 1.4E-05 | 0.00056 | VWA1, VIT, POC1A, SHOX2, SFRP2, MSX2, SCARA3, RFLNA, ZNF664-RFLNA, SMPD3, MATN4 |
| GO:0003433 | chondrocyte development involved in endochondral b | 0.00079 | 0.017 | VWA1, VIT, POC1A, SCARA3, SMPD3, MATN4 |
|  |  |  |  |  |

**Supplementary Table S3: Upregulated chondrocyte genes during *CRTAP*-null osteoblast differentiation**
