# Supplementary material for: CRTAP-Null Osteoblasts Have Increased Proliferation, Protein Secretion, and Skeletal Morphogenesis Gene Expression with Downregulation of Cellular Adhesion
Source: Cells. 2025 Mar 31;14(7):518. doi: 10.3390/cells14070518 (PMC11988066; doi:10.3390/cells14070518)
Supplement: Supplementary file 1 [file cells-14-00518-s001.zip › Supplementary table S4 Downregulated chondrocyte genes.docx]

**Supplementary Table S4: Downregulated chondrocyte genes during *CRTAP*-null osteoblast differentiation**

| **GO Pathway** | **Description** | ***p*-value** | ***p*-adjust** | **Gene ID** |
| --- | --- | --- | --- | --- |
| **DOWN - DAY 7** |  |  |  |  |
| GO:0002062 | chondrocyte differentiation | 0.0000365 | 0.00396 | OSR1, EFEMP1, BMPR1B, ADAMTS12, SCIN, SULF1, GDF6, OSR2, PTHLH, COMP, GDF5 |
| GO:0032330 | regulation of chondrocyte differentiation | 0.0000741 | 0.00647 | EFEMP1, BMPR1B, ADAMTS12, SCIN, GDF6, PTHLH, GDF5 |
| GO:0032331 | negative regulation of chondrocyte differentiation | 0.00103 | 0.0334 | EFEMP1, ADAMTS12, PTHLH, GDF5 |
| **DOWN - DAY 14** |  |  |  |  |
| GO:0032330 | regulation of chondrocyte differentiation | 0.00000533 | 0.00112 | EFEMP1, BMPR1B, ADAMTS12, FGF18, CCN2, SCIN, GDF6, SOX6, PTHLH, GREM1 |
| GO:0002062 | chondrocyte differentiation | 0.0000102 | 0.00187 | EFEMP1, BMPR1B, ADAMTS12, FGF18, COL21A1, CCN2, SCIN, GDF6, OSR2, SCX, SOX6, PTHLH, GREM1, ACAN, COMP |
